# Developing language in a developing body: genetic associations of infant gross motor behaviour and self‐care/symbolic actions with emerging language abilities

**DOI:** 10.1111/jcpp.70021

**Published:** 2025-08-31

**Authors:** Ellen Verhoef, Lucía de Hoyos, Fenja Schlag, Jeffrey van der Ven, Mitchell Olislagers, Philip S. Dale, Evan Kidd, Simon E. Fisher, Beate St Pourcain

**Affiliations:** ^1^ Language and Genetics Department Max Planck Institute for Psycholinguistics Nijmegen The Netherlands; ^2^ Department of Urology Erasmus University Medical Center, Erasmus MC Cancer Institute Rotterdam The Netherlands; ^3^ Speech & Hearing Sciences Department University of New Mexico Albuquerque NM USA; ^4^ School of Literature, Languages, and Linguistics, The Australian National University Canberra ACT Australia; ^5^ Donders Institute for Brain, Cognition and Behaviour, Radboud University Nijmegen The Netherlands; ^6^ MRC Integrative Epidemiology Unit, University of Bristol Bristol UK

**Keywords:** ALSPAC, development, genetics

## Abstract

**Background:**

Mastering gross motor abilities in early infancy and culturally defined actions (e.g. self‐care routines) in late infancy can initiate cascading developmental changes that affect language learning. Here, we adopt a genetic perspective to investigate underlying processes, implicating either shared or “gateway” mechanisms, where the latter enable children to interact with their environment.

**Methods:**

Selecting heritable traits (*h* ^2^, heritability), we studied infant gross motor (6 months) and self‐care/symbolic (15 months) skills as predictors of 10 language outcomes (15–38 months) in genotyped children from the Avon Longitudinal Study of Parents and Children (*N* ≤ 7,017). Language measures were combined into three interrelated language factors (LF) using structural equation modeling (SEM), corresponding to largely different age windows (LF_15M_, LF_24M_, LF_38M_, 51.3% total explained variance). Developmental genomic and non‐genomic relationships across measures were dissected with Cholesky decompositions using genetic‐relationship‐matrix structural equation modeling (GRM‐SEM) as part of a multivariate approach.

**Results:**

Gross motor abilities at 6 months (*h* ^2^ = 0.18 (*SE* = .06)) and self‐care/symbolic actions at 15 months (*h* ^2^ = 0.18 (*SE* = .06)) were modestly heritable, as well as the three derived language factor scores (LFS_15M_‐*h* ^2^ = 0.12 (*SE* = .05), LFS_24M_‐*h* ^2^ = 0.21 (*SE* = .06), LFS_38M_‐*h* ^2^ = 0.17 (*SE* = .05)), enabling genetic analyses. Developmental genetic models (GRM‐SEM) showed that gross motor abilities (6 months) share genetic influences with self‐care/symbolic actions (15 months, factor loading *λ*; *λ* = 0.22 (*SE* = .09)), but not with language performance (*p* ≥ .05). In contrast, genetic influences underlying self‐care/symbolic actions, independent of early gross motor skills, were related to all three language factors (LFS_15M_‐*λ* = 0.26 (*SE* = .09), LFS_24M_‐*λ* = 0.28 (*SE* = .10), LFS_38M_‐*λ* = 0.30 (*SE* = .10)). Multivariate models studying individual language outcomes provided consistent results, both for genomic and non‐genomic influences.

**Conclusions:**

Genetically encoded processes linking gross motor behaviour in young infants to self‐care/symbolic actions in older infants are different from those linking self‐care/symbolic actions to emerging language abilities. These findings are consistent with a developmental cascade where motor control enables children to engage in novel social interactions, but children's social learning abilities foster language development.

## Introduction

During the first 5 years of life, children rapidly master developmental milestones across a wide range of domains, including motor, social, cognitive and language skills (Oakes & Rakison, 2020; Zubler et al., 2022). It has been hypothesised that developmental changes in one domain can initiate a cascade of developmental changes in other domains, also known as the *developmental cascades* framework (Campos et al., 2000; Gottlieb, 1983; Thelen, 1992; Thelen & Smith, 1994). For example, observational studies provided evidence for the interrelatedness of motor, social and language development (Alcock & Connor, 2021; Libertus & Violi, 2016; Moore, Dailey, Garrison, Amatuni, & Bergelson, 2019; Oudgenoeg‐Paz, Volman, & Leseman, 2012; Quinn & Kidd, 2019; Walle & Campos, 2014), though not all studies found evidence for links between motor and language development (Moore et al., 2019).

Within the first year of life, typically developing infants acquire gross motor skills such as sitting (~6 months) and independent walking (~12 months), followed by fine motor skills such as advanced pincer grasps (~14 months; Hadders‐Algra, 2018; Landau, Smith, & Jones, 1998; Lenneberg, 1967). Motor behaviour in young infants (≤6 months) predominantly relies on the spontaneous activity of the nervous system with little adaptation due to external stimuli, referred to as the phase of primary variability (Hadders‐Algra, 2018). Subsequently, the adaptive variability of motor behaviour emerges, referred to as the phase of secondary variability (Edelman, 1993), where infants learn to use motor abilities selectively, often through social interaction such as play or observing actions from others (neural mirroring; Meltzoff, Kuhl, Movellan, & Sejnowski, 2009). Simultaneously, infants develop social abilities. For example, infants start smiling (~2 months), followed by the development of joint attention skills and engagement in interactive play (~8–9 months; Zubler et al., 2022). Thus, once early‐infant motor and social skills are mastered, children co‐develop motor and social abilities including the learning of culturally defined actions, embedded within the context of social interactions (Vygotsky, 1978). This includes both early culturally defined self‐care actions (e.g. learning how to eat with a spoon) and early symbolic gestures (e.g. pretending to pour liquid), consistent with the second variability stage for arm movements (~6–15 months; Hadders‐Algra, 2018). For example, the acquisition of learning how to eat with a spoon partially depends on the motor ability of a child to hold and move the spoon towards their mouth (Nonaka & Stoffregen, 2020). In parallel, children acquire language, with the emergence of word understanding at 6–9 months of age (Bailey & Snowling, 2002; Kuhl, 2004), the first spoken words between 10 and 15 months (Clark, 2016) and the use of two‐word combinations (also seen as the onset of grammar) from 18 to 24 months onwards (Fenson et al., 1994; Hoff, 2013).

There exist marked individual differences in the achievement of developmental milestones, which are partially attributable to genetic factors, as shown by twin and molecular studies (Austerberry, Mateen, Fearon, & Ronald, 2022; St Pourcain et al., 2014; Gui et al., 2024; Verhoef et al., 2024). Molecular studies estimate heritability from unrelated individuals utilising genome‐wide information captured by single‐nucleotide polymorphisms (SNPs) on genotyping chips (SNP‐heritability, SNP‐*h* ^2^). SNP‐based studies on early‐life developmental traits are scarce (Ronald & Gui, 2024), but recent meta‐analyses confirmed a role for genetic mechanisms contributing to the age at onset of walking (SNP‐*h* ^2^ = 24%; Gui et al., 2024) and infant/toddler vocabulary development (SNP‐*h* ^2^ = 8%–24%; Verhoef et al., 2024).

Cascading effects across developmental domains might be direct, indirect, or multidirectional (Campos et al., 2000; Iverson, 2021, 2022; Thelen & Smith, 1994). On the one hand, abilities related to motor, social, self‐care/symbolic and language skills might be interrelated because they all require motor control, manifesting as one shared underlying factor. For example, learning how to eat with a spoon partially depends on motor abilities (Nonaka & Stoffregen, 2020), gross motor skills such as more advanced sitting and walking are associated with larger expressive and receptive vocabulary sizes (Libertus & Violi, 2016; Oudgenoeg‐Paz et al., 2012; Walle & Campos, 2014), and oral motor control at 21 months (fine motor) has been linked to language production at 36 months (Alcock & Connor, 2021).

On the other hand, motor, social, self‐care/symbolic and language skills might be interlinked due to a developmental cascade (Campos et al., 2000; Gottlieb, 1983; Thelen, 1992; Thelen & Smith, 1994), in which early‐infant gross motor abilities represent a ‘gateway’ for future development. Achievement of gross motor skills during the first year of life provides infants with novel opportunities for (social) learning by enhancing their access to the environment but also by changing the nature of their social interactions (Iverson, 2021, 2022; Vygotsky, 1978, 1986). Changes in posture may, for example, enhance social interactions, illustrated by an increase in moments of shared looking and the enhanced creation of learning opportunities by caregivers once children can sit (Franchak, Kretch, & Adolph, 2018; Kretch et al., 2022). In addition, the increase in opportunities for object exploration due to advances in fine motor skills allows infants to learn about an ever‐growing variety of object properties, shaping the language input they receive from caregivers (Iverson, 2022) and playing a critical role in establishing joint attention (de Rader & Zukow‐Goldring, 2010; Nonaka & Stoffregen, 2020; Yu & Smith, 2017). While the initiation of social learning activities requires sophisticated motor control (‘gateway’), the quality of social interaction depends on different mental capacities that may also shape children's language acquisition (Campos et al., 2000; Creaghe, Quinn, & Kidd, 2021; Iverson, 2010, 2022). In other words, changes in children's motor development may increase children's learning opportunities, but children's cognitive and social abilities to use these opportunities will shape language learning through independent pathways, implicating multiple underlying factors.

Robust methodological designs are needed to model distinct developmental patterns while monitoring confounding influences and controlling for reverse causation bias (i.e. bi‐directional relations between predictor and outcome). This includes genomic approaches in combination with structural equation modelling techniques (SEM; Verhulst & Estabrook, 2012), such as a genetic‐relationship‐matrix structural equation modelling (GRM‐SEM) framework (St Pourcain et al., 2017). Implementing a developmental design through GRM‐SEM, we investigate developmental relationships linking infant motor, social and self‐care/symbolic actions (6–15 months) to infant and toddler language abilities (15–38 months) as observed for children from a UK population‐based cohort (Avon Longitudinal Study of Parents and Children, ALSPAC, *N* ≤ 7,017). Specifically, we assess the consistency of associations with developmental processes (Shapland et al., 2021), monitor confounding influences by comparing genomic and non‐genomic trait relationships and control for reverse causation bias by studying traits in developmental order.

## Methods

### Sample description and variable selection

#### Cohort information

Data were retrieved from ALSPAC (Boyd et al., 2013; Fraser et al., 2013). Ethical approval for the study was obtained from the ALSPAC Ethics and Law Committee and the Local Research Ethics Committees. Consent for biological samples has been collected in accordance with the Human Tissue Act (2004). Informed consent for the use of data collected via questionnaires and clinics was obtained from participants following the recommendations of the ALSPAC Ethics and Law Committee at the time. A detailed cohort description is provided in Appendix S1, and the study website contains details of all the data that is available through a fully searchable data dictionary and variable search tool (http://www.bristol.ac.uk/alspac/researchers/our‐data/).

#### Genetic data

Genotyping and genotype calling were performed using the Illumina HumanHap550 quad chip and Illumina GenomeStudio software. Quality control of genetic data was applied using PLINK (v1.07; Purcell et al., 2007) at both the SNP and individual level following standard procedures (Appendix S2). After quality control, 465,740 SNPs with high‐quality genetic data remained. All analyses were restricted to unrelated individuals (genomic relatedness < 0.05) with both genetic and observational data available on at least one of the variables selected (*N* ≤ 7,017). Due to different combinations of measures for each analysis, exact sample sizes may vary (reported in the legends).

#### Predictor and outcome measures

We selected measures of fine motor, gross motor and personal‐social (interactions with others and infants' ability to play, share and communicate emotions) behaviour at 6 months and self‐care/symbolic abilities at 15 months as predictors of 10 infant and toddler language outcomes (15–38 months, Table 1). All measures were assessed via parental reports using reliable and valid psychological instruments (Appendices S3 and S4). Infant fine motor, gross motor and personal‐social behaviour were assessed at 6 months of age using the corresponding scales of the Denver Developmental Screening Test (DDST; Frankenburg & Dodds, 1967). Self‐care/symbolic actions were assessed at 15 months of age using an abbreviated version of the ‘actions with objects’ section (Part II.C) of the MacArthur Communicative Development Inventory: Words and Gestures (CDI‐WG; Fenson et al., 1993; Marchman, Dale, & Fenson, 2023). Early‐life language measures, comprising both vocabulary and grammatical abilities, were assessed at 15, 24 and 38 months of age using abbreviated forms of the CDI‐WG and CDI Words and Sentences (CDI‐WS; Fenson et al., 1993; Marchman et al., 2023). Detailed measurement descriptions, including instrument reliability and validity, and questionnaire items are provided in Appendices S3 and S4.

**Table 1:** Descriptives for predictor and outcome measures

	Trait	Instrument^a^	Mean age (*SD*) in months	Mean (*SD*) *or N*_no_/*N*_yes_ ^b^	*N* (% male)
Predictors	Fine motor	DDST	6.16 (0.47)	7.74 (2.06)	5,949 (50.87%)
	Gross motor	DDST	6.16 (0.47)	5.96 (1.69)	6,334 (51.23%)
	Personal‐social	DDST	6.16 (0.47)	6.85 (1.69)	6,047 (51.10%)
	Self‐care/symbolic	CDI‐WG	15.37 (0.74)	10.99 (2.71)	6,460 (50.99%)
Outcomes	Expressive vocabulary	CDI‐WG	15.37 (0.73)	13.40 (15.44)	6,425 (51.33%)
		CDI‐WS	24.33 (0.74)	64.14 (34.99)	5,965 (51.53%)
			38.42 (0.91)	113.37 (17.29)	6,055 (51.48%)
	Morphology	CDI‐WS	24.33 (0.74)	2.37 (1.45)	5,842 (51.56%)
			38.41 (0.89)	3.84 (0.60)	5,949 (51.40%)
	Receptive vocabulary	CDI‐WG	15.37 (0.74)	72.83 (31.71)	6,488 (51.11%)
		CDI‐WS	38.42 (0.91)	109.82 (23.61)	6,044 (51.48%)
	Word form production	CDI‐WS	38.42 (0.91)	19.11 (6.46)	6,056 (51.49%)
	Combines words	CDI‐WS	24.33 (0.75)	958/4,879	5,837 (51.64%)
			38.41 (0.89)	83/5,838	5,921 (51.36%)

**Figure 1:**
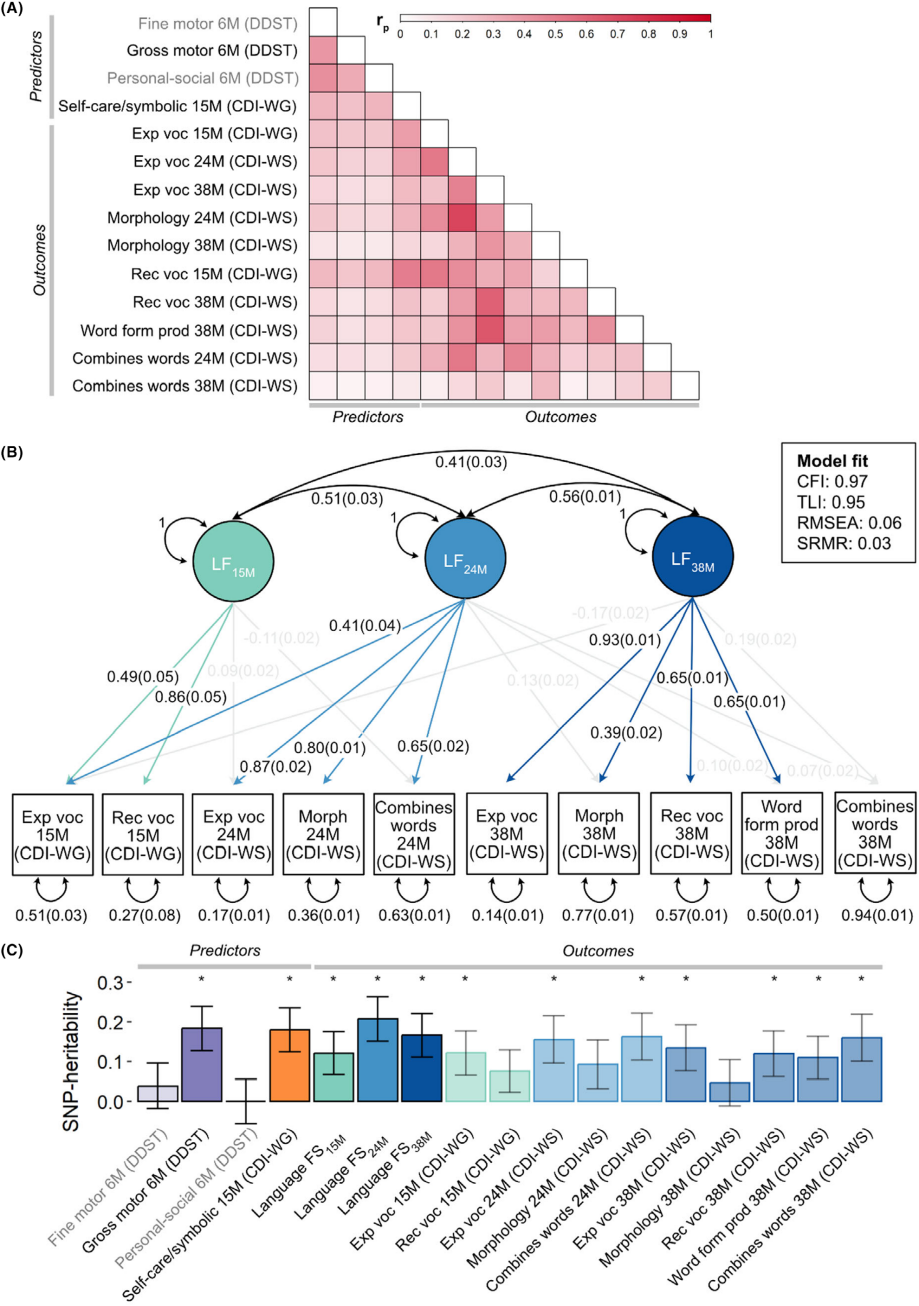
Selection of predictors and outcomes (A) Phenotypic correlations (*r*_p_) among transformed predictor and outcome measures. Estimates were obtained for transformed scores using Pearson correlation in R (R:dplyr library, v.1.1.4). Point estimates and corresponding 95%‐confidence intervals are reported in Table S1. Correlations based on untransformed measures are reported in Table S2. (B) Factor structure underlying language abilities. Confirmatory factor analysis across the 10 language abilities as estimated with lavaan following standardised parametrisation based on the full sample (*N* = 6,876). Fully standardised factor loadings (*λ*) are shown for *p* < .05 only, with meaningful factor loadings (i.e. *λ* ≥ |0.30|) depicted in colour: turquoise for LF_15m_, light blue for LF_24m_ and dark blue for LF_38m_. Observed measures are represented by squares and factors by circles. Single‐headed arrows (paths) define relationships between variables. Factors are labelled according to the age at assessment of the majority of language abilities with the strongest factor loadings. Double‐headed arrows between factors depict correlations. Double‐headed arrows at each factor reflect its variance, which has been constrained to unit variance (standardised parametrisation). Double‐headed arrows at each observed measure reflect its residual variance. (C) SNP‐heritability for predictors and outcomes (individual language measures and estimated language factor scores) estimated with GREML using GCTA software, based on directly genotyped SNPs. For predictors and individual language measures, estimates were derived for fully adjusted two‐stage transformed scores (continuous measures) or centered and scaled deviance residuals (binary measures). Bars represent standard errors. *Nominal evidence for SNP‐heritability (*p* < .05). CDI‐WG, Communicative DevelopmentInventories—Words and Gestures; CDI‐WS, Communicative Development Inventories—Words and Sentences; CFI, comparative fit index; DDST, Denver Developmental Screening Test; Exp, expressive; FS, factor score; M, months; Morph, morphology; prod, production; Rec, receptive; RMSEA, root mean square error of approximation; SNP, Single‐Nucleotide Polymorphism; SRMR, standardized root mean squared residual; TLI, Tucker–Lewis index; voc, vocabulary.

Motor, personal‐social and self‐care/symbolic actions (predictors, 6 and 15 months of age) and language measures (outcomes, 15, 24 and 38 months of age) were studied in infants and toddlers from the Avon Longitudinal Study of Parents and Children and assessed with standardised psychological instruments. Up to 7,017 children had genome‐wide genetic and developmental data on at least one of these measures available. Predictor measures with little evidence for SNP‐heritability (Figure 1C) were not included in developmental genetic models and are depicted on a grey background. CDI‐WG, Communicative Development Inventories—Words and Gestures; CDI‐WS, Communicative Development Inventories—Words and Sentences; DDST, Denver Developmental Screening Test; *N*, sample size; *SD*, standard deviation.

^a^ Abbreviated forms of the DDST, CDI‐WG and CDI‐WS were administered in ALSPAC. Details are given in the Appendices S3 and S4.

^b^ For continuous scores, the mean and standard deviation are provided; for binary scores, the number of individuals with ‘yes’ and ‘no’ answers is shown.

#### Data transformation

All variables were continuous scores, except for the measures combines words at 24 and 38 months. Continuous variable scores were adjusted for covariates and transformed using a fully adjusted two‐stage transformation (Sofer et al., 2019; Appendix S5). The two binary variables were adjusted for covariates using logistic regression. The obtained deviance residuals were centred and scaled, as applied previously (de Hoyos et al., 2024). Correlations across predictor and outcome measures, referred to here as phenotypic correlations (*r*_p_), were derived in R using Pearson correlation for transformed measures (R:dplyr library, v.1.1.4) and interpreted following recommendations (Akoglu, 2018). Phenotypic correlations for untransformed scores were estimated using Spearman correlation if one of the scores was continuous, and using Pearson correlation for two binary scores (R:dplyr library, v.1.1.4).

### Construction of language factor scores

To reduce the computational burden of downstream genetic analyses, and considering interrelations between language measures, we constructed language factor scores (LFS) across the 10 early‐life language outcomes. For this, we identified an underlying factor structure using a combination of exploratory and confirmatory factor analysis techniques, adopting a split‐half design (Appendix S6, Figure S1). LFS were estimated using the regression predictor (Devlieger, Mayer, & Rosseel, 2016; Thomson, 1934; Thurstone, 1935) (R:lavaan library, version 0.6‐12; Rosseel, 2012) for children with data on >50% of the language measures to ensure reliability (*N* = 6,433), similar to previous research (St Pourcain et al., 2011).

### SNP‐heritability and genetic correlation estimation

SNP‐*h* ^2^ of predictor and outcome measures was estimated using genome‐based restricted maximum‐likelihood (GREML) analyses (Yang, Lee, Goddard, & Visscher, 2011; Appendix S7). This method examines each pair of individuals and predicts their measurement‐score similarity based on their genetic similarity. In addition, we estimated genetic correlations (*r*_g_) using bivariate GREML (Lee, Yang, Goddard, Visscher, & Wray, 2012), reflecting the extent to which two measures share genetic influences.

### Modelling developmental genetic relationships

Using grmsem software (R:grmsem library, v1.1.2, https://gitlab.gwdg.de/beate.stpourcain/grmsem; St Pourcain et al., 2017), we fitted a Cholesky decomposition to all available data, exploiting the developmental order of the studied measures, analogous to a Direction of Causation model applied in twin research (Verhulst & Estabrook, 2012; Appendix S8). The GRM‐SEM Cholesky decomposition is a saturated model with as many latent genomic and non‐genomic factors as variables, without any restrictions on the structure (Neale, Boker, Xie, & Meas, 2006). Within a developmental context, a GRM‐SEM Cholesky decomposition investigates whether novel influences arise at each subsequent developmental timepoint, independent of previous influences, at both the genomic (A) and non‐genomic (residual, E) level. Here, A reflects additive genomic variance tagged by genotyped variants, and E reflects non‐genomic influences, including non‐genetic and non‐additive genomic variance, as well as (measurement) error (St Pourcain et al., 2017). Modelling both A and E components simultaneously allows comparing the identified structures (Appendix S9). Note that the magnitude of identifiable genomic factor loadings is bound upwards by the SNP‐*h* ^2^ of the selected measures.

To facilitate model convergence and increase interpretability, heritable predictors were selected (SNP‐*h* ^2^: *p* < .05, see Results). We fitted multiple GRM‐SEM Cholesky models using either language factor scores or individual language measures (15–38 months) as outcomes (Figure S2). We estimated genetic and residual correlations (Falconer & Mackay, 1996) using grmsem (v1.1.2; St Pourcain et al., 2017).

## Results

### Study design

To assess whether fine motor, gross motor and personal‐social behaviour in young infants (6 months) and self‐care/symbolic actions in older infants (15 months) are precursors of emerging language abilities (15–38 months), we studied parent‐reported measures and genetic information in ≤7,017 ALSPAC children (Table 1). Adopting a multi‐stage approach, we (i) studied evidence for developmental relationships, (ii) reduced the computational burden by combining language measures into factor scores, (iii) screened measures for genetic contributions and (iv) modelled underlying genomic and non‐genomic relationships using a developmental GRM‐SEM approach (Figure S2).

### Observational relationships

We observed weak‐to‐moderate phenotypic correlations across all studied developmental infant and toddler domains (Figure 1A): Motor and personal‐social skills at 6 months shared variation with self‐care/symbolic actions at 15 months (0.24 ≤ *r*_p_ < 0.29), as well as language outcomes at 15–38 months (*r*_p_ ≤ 0.28; Tables S1 and S2). Correlations with the 10 language measures were larger for self‐care/symbolic actions at 15 months (*r*_p_ ≤ 0.51) compared to the three predictor measures at 6 months (*r*_p_ ≤ 0.28), based on non‐overlapping 95%‐confidence intervals, except for the measure combines words at 38 months (Tables S1 and S2).

### Language factor identification

To reduce the computational burden of studying 10 interrelated language outcomes (0.05 ≤ *r*_p_ < 0.73, Figure 1A), we constructed factor scores. Using a combination of exploratory and confirmatory factor analysis (Methods, Appendix S10, Figures S3‐S5), we identified a correlated 3‐factor structure (*r* = 0.41 (*SE* = .03) to 0.56 (*SE* = .01)) with a good fit (Figure 1B). Together, the three factors accounted for 51.3% of the variance across the 10 language measures.

Each factor largely corresponded to a different age window (15, 24 and 38 months), suggesting developmental differences. Reporting fully standardised estimates, the first factor (LF_15M_) primarily loaded on expressive (*λ* = 0.49 (*SE* = .05)) and receptive vocabulary (*λ* = 0.86 (*SE* = .05)) assessed at 15 months. The second factor (LF_24M_) had meaningful factor loadings (*λ* ≥ |0.30|, corresponding to ~10% variance explained), on all 24‐month language measures (expressive vocabulary: *λ* = 0.87 (*SE* = .02), morphology: *λ* = 0.80 (*SE* = .01), combines words: *λ* = 0.65 (*SE* = .02)), in addition to expressive vocabulary at 15 months (*λ* = 0.41 (*SE* = .04)). The third factor (LF_38M_) explained mostly variation in measures assessed at 38 months: expressive vocabulary (*λ* = 0.93 (*SE* = .01)), receptive vocabulary (*λ* = 0.65 (*SE* = .01)), morphology (*λ* = 0.39 (*SE* = .02)) and word form production (*λ* = 0.65 (*SE* = .01)). All language measures had meaningful factor loadings, except for the measure combines words at 38 months, which may reflect the low variance of this measure (Table 1).

### Identification of heritable predictor and outcome measures

Using GREML software, we estimated the contribution of common genetic variation to predictor and outcome measures.

#### Predictors

SNP‐*h* ^2^ for gross motor skills at 6 months (SNP‐*h* ^2^ = 0.18 (*SE* = .06)) and self‐care/symbolic actions at 15 months was modest (SNP‐*h* ^2^ = 0.18 (*SE* = .06); Figure 1C). There was, however, little evidence of SNP‐*h* ^2^ for fine motor and personal‐social skills at 6 months (*p* ≥ 0.05, Figure 1C), and these measures were therefore excluded from subsequent analyses.

#### Outcomes

Language factor scores, estimated for each language factor (see above), were also modestly heritable (LFS_15M_: SNP‐*h* ^2^ = 0.12 (*SE* = .05), LFS_24M_: SNP‐*h* ^2^ = 0.21 (*SE* = .06), LFS_38M_: SNP‐*h* ^2^ = 0.17 (*SE* = .05), Figure 1C), and SNP‐*h* ^2^ was comparable to estimates obtained for individual language measures (Figure 1C). Genetic correlation analyses confirmed that derived language factor scores are largely genetically representative of individual language measures (0.54 < *r*_g_ ≤ 1.00) except for the measure combines words at 38 months (Figure S6), consistent with low loadings (*λ* ≤ 0.19) of this measure on the identified language factors (Figure 1B).

### Developmental genetic relationships

#### Links with language factor scores

**Figure 2:**
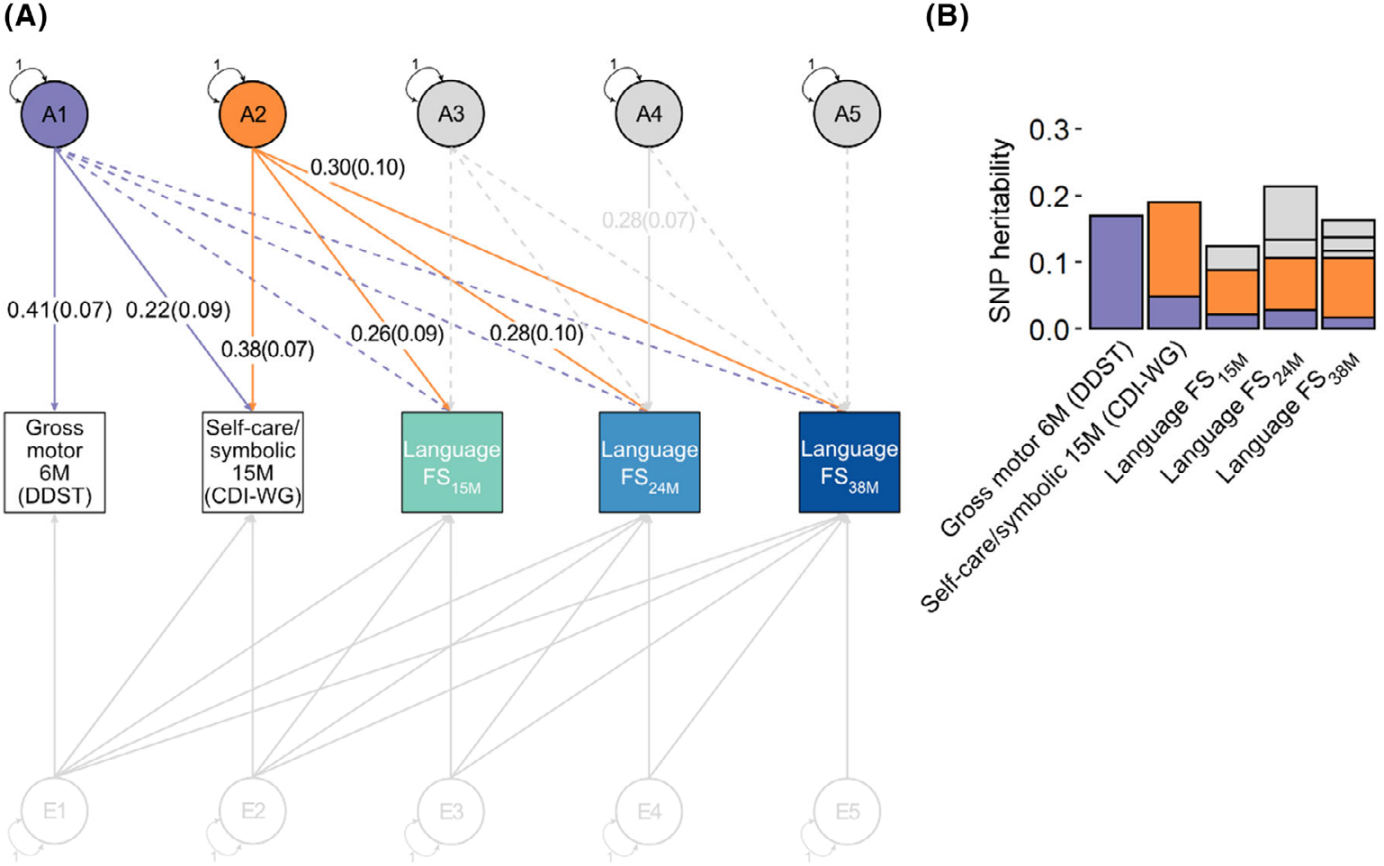
Developmental genetic relationships of gross motor (6 months) and self‐care/symbolic actions (15 months) with language factor scores (A) Path diagram with standardised path coefficients and corresponding standard errors for a Cholesky decomposition of gross motor skills (6 months), self‐care/symbolic actions (15 months) and the three estimated language factor scores (Language FS_15m_, Language FS_24m_ and Language FS_38m_), in that order. Analyses were carried out with genetic‐relationship‐matrix structural equation modelling (GRM‐SEM, grmsem v.1.1.2 software) using all available observations for children across development (*N* ≤ 6,975). Observed measures are represented by squares and factors by circles. Single‐headed arrows (paths) define relationships between variables. Latent variables were standardised to unit variance (double‐headed arrows). Paths with a path coefficient of *p* < .05 are indicated by solid lines, while dashed lines indicate paths with a path coefficient of *p* ≥ .05. Genetic relationships between predictor and outcome variables are depicted in colour, others in grey. Full information on all path coefficients and their standard errors can be found in Table S3. (B) SNP‐heritability attributable to genomic factors modelled in (A). CDI‐WG, Communicative Development Inventories—Words and Gestures; DDST, Denver Developmental Screening Test; FS, factor score; M, months; SNP, Single‐Nucleotide Polymorphism.

To investigate whether observational relationships are (partially) due to shared genetic influences, we fitted a GRM‐SEM Cholesky decomposition model to gross motor skills at 6 months, self‐care/symbolic actions at 15 months and the three language factor scores (LFS_15M_, LFS_24M_ and LFS_38M_, in this order, Figure 2, Table S3). Genetic influences identified for gross motor skills (termed A1, *λ* = 0.41 (*SE* = .07)) were also related to self‐care/symbolic actions (*λ* = 0.22 (*SE* = .09)). There was, however, little evidence for the association of A1 with any of the language factor scores (*p* ≥ .05). In contrast, genetic influences identified for self‐care/symbolic actions in older infants that are independent of gross motor behaviour in younger infants (termed A2, *λ* = 0.38 (*SE* = .07)) were associated with concurrent and toddler language development (LFS_15M_: *λ* = 0.26 (*SE* = .09), LFS_24M_: *λ* = 0.28 (*SE* = .10), LFS_38M_: *λ* = 0.30 (*SE* = .10)). There was little evidence (*p* ≥ .05) for genetic influences on language factor scores beyond those already captured by the studied predictors (A3–A5), except for LFS_24M_ (A4, *λ* = 0.28 (*SE* = .07)).

#### Links with individual language measures

**Figure 3:**
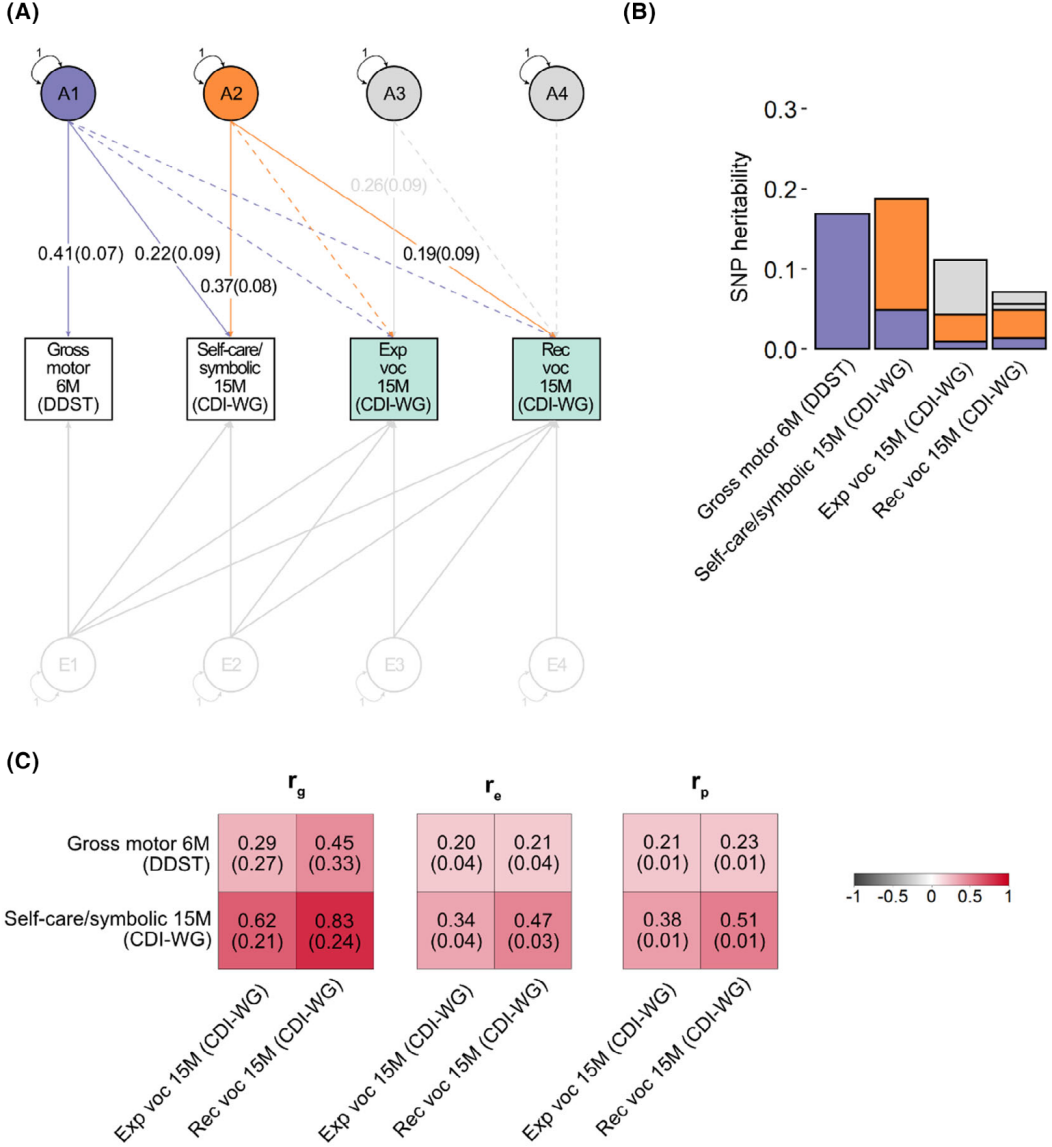
Developmental genetic relationships of infant gross motor (6 months) and self‐care/symbolic actions (15 months) with language measures (15 months) Cholesky decomposition of gross motor skills (6 months), self‐care/symbolic actions (15 months), expressive and receptive vocabulary size (both at 15 months), in that order. Analyses were carried out with genetic‐relationship matrix structural equation modelling (GRM‐SEM, grmsem v.1.1.2 software) using all available observations for children across development (*N* ≤ 6,908). (A) Path diagram with standardised path coefficients and corresponding standard errors for a Cholesky decomposition. Observed measures are represented by squares and factors by circles. Single‐headed arrows (paths) define relationships between variables. Latent variables were standardised to unit variance (double‐headed arrows). Paths with a path coefficient of *p* < .05 are indicated by solid lines, while dashed lines indicate paths with a path coefficient of *p* ≥ .05. Full information on all path coefficients and their standard errors can be found in Table S4. (B) SNP‐heritability attributable to genomic factors modelled in (A). (C) genetic (*r*_g_), residual/non‐genomic (*r*_e_) and phenotypic (*r*_p_) correlation patterns of gross motor skills (6 months) and self‐care/symbolic actions (15 months) with language measures at 15 months, as derived from the Cholesky decomposition model (shown in A). Phenotypic correlations (*r*_p_) were estimated with Pearson correlation. Standard errors are shown in parentheses. CDI‐WG, Communicative Development Inventories—Words and Gestures; DDST, Denver Developmental Screening Test; Exp, expressive; M, months; Rec, receptive; SNP, Single‐Nucleotide Polymorphism; voc, vocabulary.

**Figure 4:**
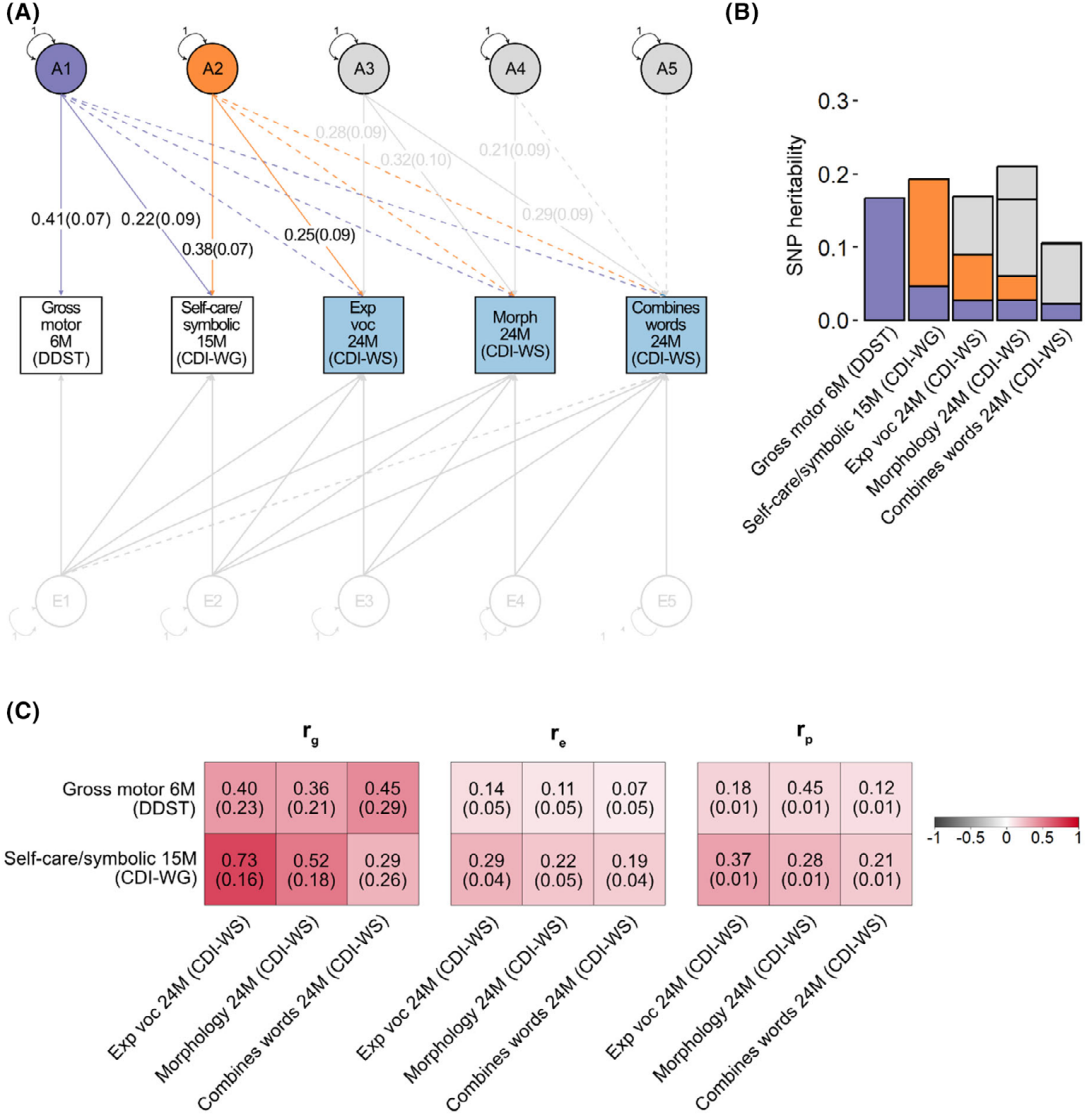
Developmental genetic relationships of infant gross motor (6 months) and self‐care/symbolic actions (15 months) with language measures (24 months) Cholesky decomposition of gross motor skills (6 months), self‐care/symbolic actions (15 months), expressive vocabulary size (24 months), morphology (24 months) and the use of word combinations (24 months), in that order. Analyses were carried out with genetic‐relationship‐matrix structural equation modelling (GRM‐SEM, grmsem v.1.1.2 software) using all available observations for children across development (*N* ≤ 6,976). (A) Path diagram with standardised path coefficients and corresponding standard errors for a Cholesky decomposition. Observed measures are represented by squares and factors by circles. Single‐headed arrows (paths) define relationships between variables. Latent variables were standardised to unit variance (double‐headed arrows). Paths with a path coefficient of *p* < .05 are indicated by solid lines, while dashed lines indicate paths with a path coefficient of *p* ≥ .05. Full information on all path coefficients and their standard errors can be found in Table S5. (B) SNP‐heritability attributable to genomic factors modelled in (A). (C) Genetic (*r*_g_), residual/non‐genomic (*r*_e_) and phenotypic (*r*_p_) correlation patterns of gross motor skills (6 months) and self‐care/symbolic actions (15 months) with language measures at 24 months, as derived from the Cholesky decomposition model (shown in a). Phenotypic correlations (*r*_p_) were estimated with Pearson correlation. Standard errors are shown in parentheses. CDI‐WS, Communicative Development Inventories—Words and Sentences; DDST, Denver Developmental Screening Test; Exp, expressive; M, months; SNP, single‐nucleotide polymorphism; voc, vocabulary.

**Figure 5:**
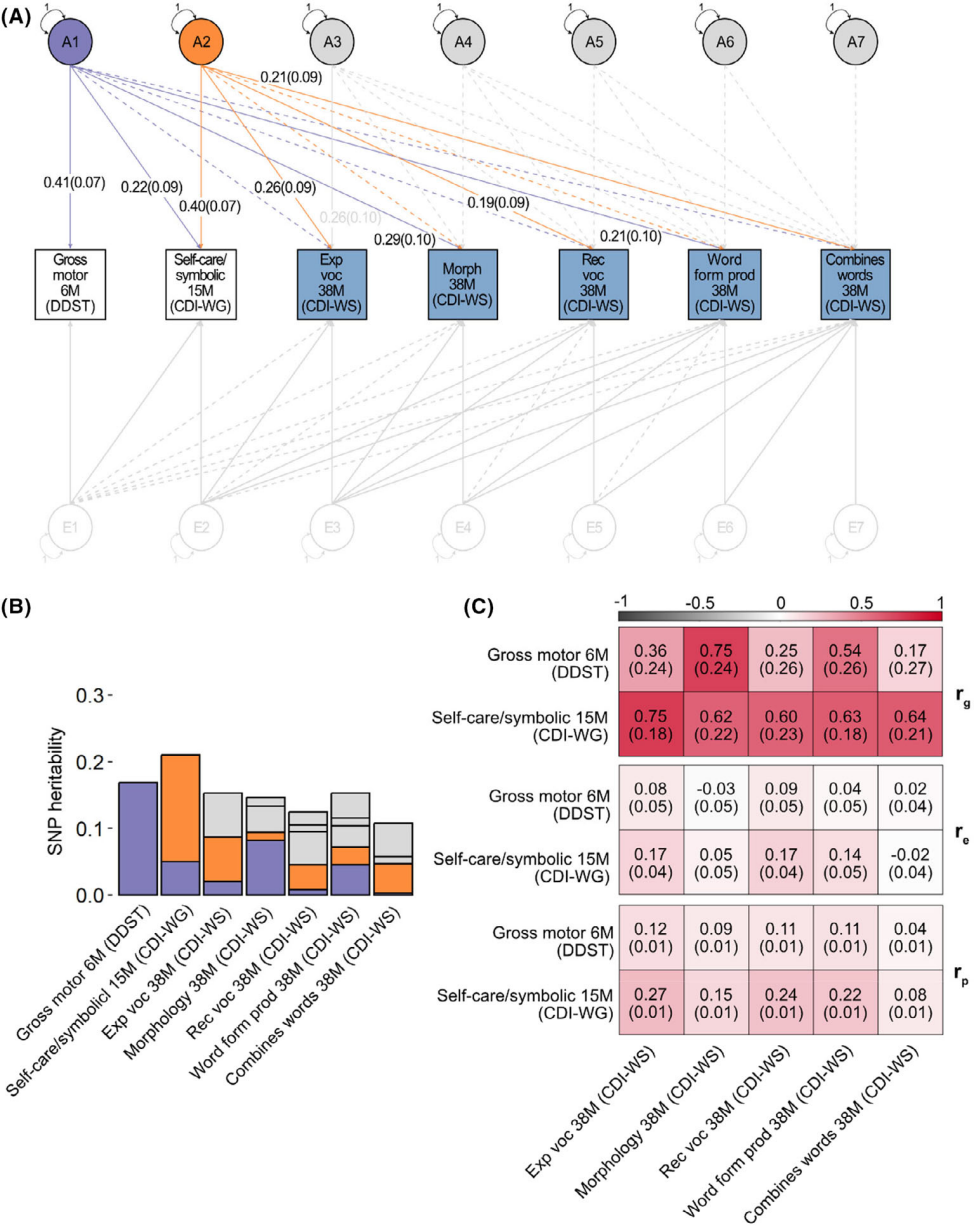
Developmental genetic relationships of infant gross motor (6 months) and self‐care/symbolic actions (15 months) with language measures (38 months) Cholesky decomposition of gross motor skills (6 months), self‐care/symbolic actions (15 months), expressive vocabulary size (38 months), morphology (38 months), receptive vocabulary size (38 months), word form production (38 months) and the use of word combinations (38 months), in that order. Analyses were carried out with genetic‐relationship‐matrix structural equation modelling (GRM‐SEM, grmsem v.1.1.2 software) using all available observations for children across development (*N* ≤ 6,978). (A) Path diagram with standardised path coefficients and corresponding standard errors for a Cholesky decomposition. Observed measures are represented by squares and factors by circles. Single‐headed arrows (paths) define relationships between variables. Latent variables were standardised to unit variance (double‐headed arrows). Paths with a path coefficient of *p* < .05 are indicated by solid lines, while dashed lines indicate paths with a path coefficient of *p* ≥ .05. Full information on all path coefficients and their standard errors can be found in Table S6. (B) SNP‐heritability attributable to genomic factors modelled in (A). (C) Genetic (*r*_g_), residual/non‐genomic (*r*_e_) and phenotypic (*r*_p_) correlation patterns of gross motor skills (6 months) and self‐care/symbolic actions (15 months) with language measures at 38 months, as derived from the Cholesky decomposition model (shown in A). Phenotypic correlations (*r*_p_) were estimated with Pearson correlation. Standard errors are shown in parentheses. CDI‐WS, Communicative Development Inventories—Words and Sentences; DDST, Denver Developmental Screening Test; Exp, expressive; M, months; Rec, receptive; SNP, single‐nucleotide polymorphism; voc, vocabulary.

While genetic analyses of language factor scores represent broadly‐shared genetic relationships (see above, Figure S6), they do not fully capture the variance of each individual language measure (e.g. combines words at 38 months). Therefore, we conducted three further age‐specific GRM‐SEM Cholesky decomposition models as part of follow‐up analyses (Figures 3, 4, 5). Each of these models included gross motor skills at 6 months and self‐care/symbolic actions at 15 months as predictors along with the set of language measures with the largest loadings for each identified language factor (Figure 1B).

Findings from age‐specific GRM‐SEM models (Figures 3, 4, 5) were highly similar to those including language factor scores (Figure 2). In particular, genetic influences identified for infant self‐care/symbolic actions independent of earlier gross motor abilities (A2) were related to language measures across early development (i.e. 15–38 months). Specifically, for language assessments at 15 months, A2 was associated with receptive vocabulary (*λ* = 0.19 (*SE* = .09), Figure 3A,B, Table S4). At 24 months of age, A2 accounted for genetic variation in expressive vocabulary size (*λ* = 0.25 (*SE* = .09), Figure 4A,B, Table S5), and at 38 months of age, A2 was linked to expressive vocabulary (*λ* = 0.26 (*SE* = .09)), receptive vocabulary (*λ* = 0.19 (*SE* = .09)) and the use of word combinations (*λ* = 0.21 (*SE* = .09), Figure 5A,B, Table S6). In contrast, there was little evidence (*p* ≥ .05) for genetic association between gross motor abilities at 6 months of age (A1) and language abilities at 15 and 24 months of age (Figures 3 and 4, Tables S4 and S5). However, at 38 months of age, A1 was associated with both morphology (*λ* = 0.29 (*SE* = .10)) and word form production (*λ* = 0.21 (*SE* = .10)), suggesting shared influences between gross motor, self‐care/symbolic and language development as captured by a single genomic factor (Figure 5A,B, Table S6). Across all ages, we observed evidence for genetic influences contributing to individual language measures beyond those captured by the studied predictors, suggesting language‐specific contributions (Figures 3, 4, 5).

#### Sensitivity analyses

Adopting an alternative model specification to derive language factor scores provided near‐identical results, underlining the robustness of our findings (Appendix S6, Table S7, Figures S7 and S8). For all developmental genetic models, GREML and GRM‐SEM provided consistent SNP‐*h* ^2^ estimates (Table S8).

#### Comparisons of genomic and non‐genomic influences

Concordance of genomic (A) and non‐genomic (E) factor structures indicates the consistency of associations with developmental relationships (Cheverud, 1988; Sanderson et al., 2022; Shapland et al., 2021; Appendix S9). Here, we focused on models studying individual language outcomes, that is, models with full genomic and non‐genomic variance information (unlike language factor scores). The identified genomic structures linking gross motor and self‐care/symbolic actions to individual language outcomes were consistent with both non‐genomic and phenotypic relationships, except for links with morphology and word form production at 38 months (Figures 3, 4, 5). For the latter two measures, despite genetic overlap, there was little evidence for non‐genomic correlations with infant gross motor abilities (Figure 5C), making direct relationships less likely.

## Discussion

Investigating developmental relationships linking gross motor behaviour in young infants and self‐care/symbolic actions in older infants to emerging language abilities, as observed in up to 7,017 unrelated children, this study provides evidence for at least two independent underlying genomic factors. While genetic influences underlying early infant gross‐motor behaviour were largely shared with subsequent self‐care/symbolic actions, these influences contributed little to language performance in infancy and toddlerhood, except for higher‐order rule‐based grammatical abilities. Instead, genetic influences unique to self‐care/symbolic actions (and independent of gross motor skills) were interlinked with the majority of language outcomes.

Genetic influences contributing to early infant gross motor skills were moderately related to self‐care/symbolic actions at 15 months, consistent with observational relationships. Such influences could potentially encode processes related to muscle control and executive functioning (Zeng, Hu, & Wang, 2023) that may also affect culturally‐defined self‐care/symbolic actions. In addition to shared genomic influences, there was evidence for shared non‐genomic influences with a similar direction of effect. Consistent genomic and non‐genomic relationships support developmental links between early infant gross motor skills and subsequent self‐care/symbolic actions, in line with shared aetiological mechanisms between primary and secondary motor abilities (Hadders‐Algra, 2018). However, genetic influences underlying gross motor skills at 6 months showed little overlap with subsequent vocabulary and lower‐level grammatical skills, except for association with higher‐order rule‐based grammatical skills (morphological and word form production abilities at 38 months). Despite genetic links, our findings are unlikely to capture direct effects of early infant motor control on later language, considering diverging genomic and non‐genomic correlation patterns (Figure 5C), although they do not preclude indirect effects. Thus, despite phenotypic correlations of early‐life gross motor skills with language outcomes consistent with prior research (Libertus & Violi, 2016; Oudgenoeg‐Paz et al., 2012; Walle & Campos, 2014), there was limited evidence for a single, shared genomic factor contributing to this relationship.

Genetic influences underlying self‐care/symbolic actions independent of gross motor skills were associated with concurrent and toddler language performance, especially vocabulary size and lower‐level grammatical skills. This genetic link is consistent with findings from an independent study in autistic individuals, showing that children's developmental age to self‐feed with a spoon was genetically more similar to language‐related outcomes than to children's age of crawling (de Hoyos et al., 2024). Furthermore, we found concordant genomic and non‐genomic structures between infant self‐care/symbolic and multiple concurrent/toddler vocabulary and grammatical skills, consistent with developmental links. Thus, in line with a developmental cascade (Campos et al., 2000; Gottlieb, 1983; Thelen, 1992; Thelen & Smith, 1994), genetically encoded processes linking gross motor behaviour in young infants to self‐care/symbolic actions in older infants were independent from those linking infant self‐care/symbolic actions to concurrent and later language abilities.

Following such a cascade, early infant motor abilities may represent a ‘gateway’ for future development. While participation in and the acquisition of culturally defined actions with objects, for example related to self‐care or symbolic actions, require adaptive variability of motor behaviour, the resulting enhanced social activity may also affect children's language learning (Campos et al., 2000; Creaghe et al., 2021; Iverson, 2010, 2022; Kretch et al., 2022), implicating two different mechanisms: (i) Mastering self‐care/symbolic activities that require motor control may enhance children's social activity with caregivers, providing more learning opportunities. (ii) Self‐care/symbolic actions are the consequence of and evidence for social learning, an ability that is crucial for mastering language, consistent with intergenerational cultural transmission and socio‐cultural approaches to development (Vygotsky, 1978, 1986). Thus, our findings support observational reports showing that interaction acts as a key driver of early language growth (Donnelly & Kidd, 2021) and that vocabulary scores have stronger and more robust links with the onset of pointing than with the onset of walking (Moore et al., 2019). Together, our results underline the importance of social and symbolic aspects for language learning.

In addition to social learning, genetic influences identified for self‐care/symbolic actions at 15 months that are independent of gross motor skills at 6 months may encode further processes, including fine motor control. Multiple self‐care and symbolic actions rely on fine motor control (e.g. putting on a necklace) that may differ in underlying development processes from gross motor skills (Hegemann et al., 2024) and have different contributions to language development (Gonzalez, Alvarez, & Nelson, 2019). While fine motor abilities at the age of 15 months rely on experience‐based adaptations of behaviour, especially through social interactions, early infant gross motor abilities (≤6 months) involve predominantly (but not exclusively) spontaneous activities of the nervous system (Hadders‐Algra, 2018). It is, however, not yet possible to identify the genetic mechanisms captured by the identified genomic factors, similar to multivariate twin analysis. Also, the assessment of shared genetic links with processes of oral motor development underlying speech in older infants is hampered, given the paucity of data. The interpretation of genomic and non‐genomic correlations, therefore, relies on their consistency, and the alignment with previous research and theoretical notions.

This study has several strengths and limitations. First, adopting a genetic (i.e. GRM‐SEM) modelling approach with a developmental design, we dissect patterns of genomic and non‐genomic association and evaluate their consistency with developmental processes (Shapland et al., 2021) while monitoring confounding influences and bias. The concordant genomic and non‐genomic structures for language assessments at 15, 24 and 38 months suggest that our findings are unlikely to be driven by measurement error or non‐genomic/residual confounding. The temporal order of variables within our study (applicable to 8/10 studied language measures) largely precludes that the identified relationships are due to reverse causation (i.e. variation in language abilities affecting self‐care/symbolic actions; Pingault et al., 2018). Thus, it is likely that infant motor achievements foster subsequent language acquisition by affecting children's learning environment, including their social interactions (Iverson, 2022). Second, as SNP‐*h* ^2^ estimates for all studied traits were modest (5%–21%), corresponding path coefficients identified in developmental genetic models were small, limiting the interpretation of variance components. Third, the low heritability of fine motor and personal‐social behaviour at 6 months prevented the inclusion of these measures as predictors in our genetic analyses although they may still contribute to later self‐care, symbolic and language development. Fourth, the presented findings are based on a single, English‐speaking, Western European cohort and warrant replication in independent datasets, ideally studying individuals from different linguistic and cultural backgrounds. Future, more powerful genetic studies on early‐life developmental traits across different cultures are required to further investigate genetic components underlying proposed developmental cascades and causal mechanisms.

Taken together, our findings suggest that, consistent with a developmental cascade, there are distinct processes linking gross motor to self‐care/symbolic actions versus self‐care/symbolic actions to language abilities: While genetic influences related to gross motor development in early infancy may lay the foundation for sensorimotor developments that increase children's engagement in social learning activities, the majority of genetic links with concurrent and toddler language abilities are explained by an independent genomic factor identified for culturally defined self‐care/symbolic actions during later infancy.

## Ethical considerations

Ethical approval for the study was obtained from the ALSPAC Ethics and Law Committee and the Local Research Ethics Committees. Consent for biological samples has been collected in accordance with the Human Tissue Act (2004). Informed consent for the use of data collected via questionnaires and clinics was obtained from participants following the recommendations of the ALSPAC Ethics and Law Committee at the time. A detailed cohort description is provided in Appendix S1, and the study website contains details of all the data that are available through a fully searchable data dictionary and variable search tool (http://www.bristol.ac.uk/alspac/researchers/our‐data/).

**Key points**

- The acquisition of developmental milestones in young children is a complex and intertwined process that is characterised by cascading progress across multiple domains.
- Gross motor abilities in young infants are genetically linked to self‐care/symbolic actions in older infants and, independently, self‐care/symbolic actions genetically overlap with infant/toddler language performance. These findings support the theory that early motor achievements provide a ‘gateway’ for children to engage with their environment, enabling social learning opportunities and thereby fostering language development.
- Culturally‐defined actions, such as self‐care and symbolic actions, play a key role in language learning, reflecting a combination of motor and social learning that synergistically enhances language development.
- Enabling and enhancing social learning situations may support children's language acquisition during infancy and toddlerhood.

## Supporting information


**Appendix S1.** Avon Longitudinal Study of Parents and Children.
**Appendix S2.** Quality control of genotyping data.
**Appendix S3.** Measurement description.
**Appendix S4.** Description of psychological instruments.
**Appendix S5.** Data transformation for continuous scores.
**Appendix S6.** Construction of language factor scores – methods.
**Appendix S7.** Genome‐based Restricted Maximum‐Likelihood analyses.
**Appendix S8.** The Cholesky decomposition as a multivariate form of a Direction of Causation model.
**Appendix S9.** Evaluating consistency with developmental relationships.
**Appendix S10.** Construction of language factor scores – results.
**Table S1.** Phenotypic correlations between transformed measures.
**Table S2.** Phenotypic correlations between untransformed measures.
**Table S3.** Developmental genetic relationships of gross motor behaviour (6 months) and self‐care/symbolic actions (15 months) with language factor scores.
**Table S4.** Developmental genetic relationships of gross motor behaviour (6 months) and self‐care/symbolic actions (15 months) with language measures (15 months).
**Table S5.** Developmental genetic relationships of gross motor behaviour (6 months) and self‐care/symbolic actions (15 months) with language measures (24 months).
**Table S6.** Developmental genetic relationships of gross motor behaviour (6 months) and self‐care/symbolic actions (15 months) with language measures (38 months).
**Table S7.** Developmental genetic relationships of gross motor behaviour (6 months) and self‐care/symbolic actions (15 months) with language factor scores (sensitivity analysis).
**Table S8.** GREML and GRM‐SEM SNP‐heritability.
**Figure S1.** Exploratory and confirmatory factor analysis design.
**Figure S2.** Study design.
**Figure S3.** EFA: Phenotypic correlations among language measures.
**Figure S4.** Estimated number of common factors underlying language measures.
**Figure S5.** Confirmatory factor analysis model (split‐half design).
**Figure S6.** Genetic correlations of language factor scores with individual language measures.
**Figure S7.** Factor structure underlying language abilities (sensitivity analysis).
**Figure S8.** Distribution of derived language factor scores.

## Data Availability

The data used in this work are available through a fully searchable data dictionary (http://www.bris.ac.uk/alspac/researchers/data‐access/data‐dictionary/). Access to ALSPAC data can be obtained following the ALSPAC data access policy (http://www.bristol.ac.uk/alspac/researchers/access/). All analyses were performed using freely accessible software, and requests for analysis details, including scripts, can be sent via email to the corresponding author.
