# Supplementary material for: Developing language in a developing body: genetic associations of infant gross motor behaviour and self‐care/symbolic actions with emerging language abilities
Source: J Child Psychol Psychiatry. 2025 Aug 31;67(1):41–54. doi: 10.1111/jcpp.70021 (PMC12699111; doi:10.1111/jcpp.70021)
Supplement: Supplementary file 1 — Appendix S1. Avon Longitudinal Study of Parents and Children. Appendix S2. Quality control of genotyping data. Appendix S3. Measurement description. Appendix S4. Description of psychological instruments. Appendix S5. Data transformation for continuous scores. Appendix S6. Construction of language factor scores – methods. Appendix S7. Genome‐based Restricted Maximum‐Likelihood analyses. Appendix S8. The Cholesky decomposition as a multivariate form of a Direction of Causation model. Appendix S9. Evaluating consistency with developmental relationships. Appendix S10. Construction of language factor scores – results. Table S1. Phenotypic correlations between transformed measures. Table S2. Phenotypic correlations between untransformed measures. Table S3. Developmental genetic relationships of gross motor behaviour (6 months) and self‐care/symbolic actions (15 months) with language factor scores. Table S4. Developmental genetic relationships of gross motor behaviour (6 months) and self‐care/symbolic actions (15 months) with language measures (15 months). Table S5. Developmental genetic relationships of gross motor behaviour (6 months) and self‐care/symbolic actions (15 months) with language measures (24 months). Table S6. Developmental genetic relationships of gross motor behaviour (6 months) and self‐care/symbolic actions (15 months) with language measures (38 months). Table S7. Developmental genetic relationships of gross motor behaviour (6 months) and self‐care/symbolic actions (15 months) with language factor scores (sensitivity analysis). Table S8. GREML and GRM‐SEM SNP‐heritability. Figure S1. Exploratory and confirmatory factor analysis design. Figure S2. Study design. Figure S3. EFA: Phenotypic correlations among language measures. Figure S4. Estimated number of common factors underlying language measures. Figure S5. Confirmatory factor analysis model (split‐half design). Figure S6. Genetic correlations of language factor scores with individual language measures. [file JCPP-67-41-s001.docx]

**Supporting Information**

Developing language in a developing body:
Genetic associations of infant gross motor behaviour and self-care/symbolic actions
with emerging language abilities

**Table of Contents**

[Supplementary Notes 3](#_Toc195989163)

[Appendix S1: Avon Longitudinal Study of Parents and Children 3](#_Toc195989164)

[Appendix S2: Quality control of genotyping data 5](#_Toc195989165)

[Appendix S3: Measurement description 6](#_Toc195989166)

[Appendix S4: Description of psychological instruments 11](#_Toc195989167)

[Appendix S5: Data transformation for continuous scores 13](#_Toc195989168)

[Appendix S6: Construction of language factor scores - methods 14](#_Toc195989169)

[Appendix S7: Genome-based Restricted Maximum-Likelihood analyses 16](#_Toc195989170)

[Appendix S8: The Cholesky decomposition as a multivariate form of a Direction of Causation model 17](#_Toc195989171)

[Appendix S9: Evaluating consistency with developmental relationships 18](#_Toc195989172)

[Appendix S10: Construction of language factor scores - results 19](#_Toc195989173)

[Supplementary Tables 20](#_Toc195989174)

[Table S1: Phenotypic correlations between transformed measures 21](#_Toc195989175)

[Table S2: Phenotypic correlations between untransformed measures 24](#_Toc195989176)

[Table S3: Developmental genetic relationships of gross motor behaviour (6 months) and self-care/symbolic actions (15 months) with language factor scores 27](#_Toc195989177)

[Table S4: Developmental genetic relationships of gross motor behaviour (6 months) and self-care/symbolic actions (15 months) with language measures (15 months) 28](#_Toc195989178)

[Table S5: Developmental genetic relationships of gross motor behaviour (6 months) and self-care/symbolic actions (15 months) with language measures (24 months) 29](#_Toc195989179)

[Table S6: Developmental genetic relationships of gross motor behaviour (6 months) and self-care/symbolic actions (15 months) with language measures (38 months) 30](#_Toc195989180)

[Table S7: Developmental genetic relationships of gross motor behaviour (6 months) and self-care/symbolic actions (15 months) with language factor scores (sensitivity analysis) 32](#_Toc195989181)

[Table S8: GREML and GRM-SEM SNP-heritability 34](#_Toc195989182)

[Supplementary Figures 35](#_Toc195989183)

[Figure S1: Exploratory and confirmatory factor analysis design 36](#_Toc195989184)

[Figure S2: Study design 37](#_Toc195989185)

[Figure S3: EFA: Phenotypic correlations among language measures 38](#_Toc195989186)

[Figure S4: Estimated number of common factors underlying language measures 39](#_Toc195989187)

[Figure S5: Confirmatory factor analysis model (split-half design) 40](#_Toc195989188)

[Figure S6: Genetic correlations of language factor scores with individual language measures 41](#_Toc195989189)

[Figure S7: Factor structure underlying language abilities (sensitivity analysis) 42](#_Toc195989190)

[Figure S8: Distribution of derived language factor scores 44](#_Toc195989191)

[Supplementary References 45](#_Toc195989192)

# **Supplementary Notes**

## **Appendix S1: Avon Longitudinal Study of Parents and Children**

Pregnant women resident in Avon, UK with expected dates of delivery between 1st April 1991 and 31st December 1992 were invited to take part in the study. 20,248 pregnancies have been identified as being eligible and the initial number of pregnancies enrolled was 14,541. Of the initial pregnancies, there was a total of 14,676 foetuses, resulting in 14,062 live births and 13,988 children who were alive at 1 year of age. When the oldest children were approximately 7 years of age, an attempt was made to bolster the initial sample with eligible cases who had failed to join the study originally. As a result, when considering variables collected from the age of seven onwards (and potentially abstracted from obstetric notes) there are data available for more than the 14,541 pregnancies mentioned above: The number of new pregnancies not in the initial sample (known as Phase I enrolment) that are currently represented in the released data and reflecting enrolment status at the age of 24 is 906, resulting in an additional 913 children being enrolled (456, 262 and 195 recruited during Phases II, III and IV respectively). The phases of enrolment are described in more detail in the cohort profile paper and its update (Boyd et al., 2013; Fraser et al., 2013). The total sample size for analyses using any data collected after the age of seven is therefore 15,447 pregnancies, resulting in 15,658 foetuses. Of these 14,901 children were alive at 1 year of age.

Of the original 14,541 initial pregnancies, 338 were from a woman who had already enrolled with a previous pregnancy, meaning 14,203 unique mothers were initially enrolled in the study. As a result of the additional phases of recruitment, a further 630 women who did not enrol originally have provided data since their child was 7 years of age. This provides a total of 14,833 unique women (G0 mothers) enrolled in the Avon Longitudinal Study of Parents and Children (ALSPAC) as of September 2021.

G0 partners were invited to complete questionnaires by the mothers at the start of the study and they were not formally enrolled at that time. 12,113 G0 partners have been in contact with the study by providing data and/or formally enrolling when this started in 2010. 3,807 G0 partners are currently enrolled.

## **Appendix S2: Quality control of genotyping data**

Quality control of genetic data was applied using PLINK (v1.07)(Purcell et al., 2007) at both the SNP and individual level following standard procedures. Individuals were excluded in case of gender mismatch between reported and genetic sex, >3% missing SNP information, non-European genetic ancestry, or interindividual relatedness (genomic relatedness>0.05). SNPs were excluded if they had a low call rate (<99%), were rare (<1%) and/or deviated from Hardy-Weinberg equilibrium (*P*<5x10^-7^).

## **Appendix S3: Measurement description**

Motor, personal-social, self-care/symbolic and language development during infancy and toddlerhood were assessed based on abbreviated versions of the Denver Developmental Screening Test (DDST)(Frankenburg & Dodds, 1967), the MacArthur CDI: Words & Gestures (CDI-WG), and the MacArthur CDI: Words & Sentences (CDI-WS)(Fenson et al., 1993; Marchman, Dale, & Fenson, 2023). The measures are described in detail below, and validity and reliability of instruments is reported in Appendix S4.

Motor skills (6 months of age)

*Gross motor skills*

At six months of age, parents were asked about their child’s gross motor achievements, according to 13 items of the Denver Developmental Screening Test (DDST)(Frankenburg & Dodds, 1967). Each item (see below) was coded as zero if a child had not yet started the activity, and coded as one if a child had done the activity once or twice, or often. Next, a summary score across all 13 items was created, reflecting gross motor development.

DDST items: child keeps head steady when sitting, child lifts chest and shoulders when on front, child can roll over, child bears weight on legs when upright, child can sit up with no support, child can stand whilst holding something, child can pull self-up by holding something, child can sit from standing position, child can walk when holding something, child lifts head when on front, child can stand with no support, child can stop and stand again when upright, child can crawl on hands and knees.

*Fine motor skills*

Parents were also asked about their child’s fine motor abilities at six months of age. These were assessed according to 11 items from the DDST (Frankenburg & Dodds, 1967) (see below) that were coded similarly to the gross motor items: a score of zero was awarded if a child had not yet started the activity, and a score of one was awarded if the child had done the activity once or twice, or often. Summing the 11 fine motor items resulted in a score reflecting fine motor development.

DDST items: child can put hands together, child can hold a rattle, child can focus eyes on small objects, child reaches for objects, child looks for hidden objects, child can pick up two things at once when sitting, child can pick up small objects, child can pass objects from hand to hand, child can bang held objects together, child grabs objects with whole hand, child picks object up with four fingers and thumb.

Personal-social skills (6 months of age)

Ten items from the DDST (Frankenburg & Dodds, 1967) were utilised to assess children’s personal-social development at six months of age (see below). For each item a score of zero was awarded if a child had not yet started this behaviour, while a score of one was awarded if a child had expressed this behaviour once or twice, or often. A summary score across the ten items was then created to reflect personal-social development at six months of age.

DDST items: child looks at older people’s faces, child smiles when smiled at, child does not want toys taken away, child can feed self with rusk or similar, child tries for out of reach toys, child is shy with strangers, child plays peek-a-boo, child plays pat-a-cake, child can drink from a cup, child shows what is wanted without crying.

Actions with objects (15 months of age)

Culturally-defined actions with objects were assessed at 15 months of age, using 16 out of 17 items from Part II.C of the MacArthur CDI:Words & Gestures (CDI-WG)(Fenson et al., 2000) that are related to self-care and symbolic gesture (see below). A child was awarded a score of zero for each item if he/she did not show this behaviour, and a score of one if the child tried or had already acquired the skill. A summary score was created by summing across all items.

CDI-WG items: child eats with spoon or fork, child drinks liquid from a cup, child combs or brushes own hair, child brushes teeth, child wipes face or hands with a towel, child puts a hat on, child puts shoe or sock on, child puts necklace, bracelet or watch on, child puts head on its hands and shuts its eyes as if asleep, child blows as if something is hot, child holds and flies a plane, child holds a phone to its ear, child sniffs flowers, child pushes car or truck, child pretends to pour liquid, child stirs pretend liquid in a cup or a pan.

Language abilities (15, 24 and 38 months of age)

Expressive and receptive vocabulary size were assessed using abbreviated versions of the CDI-WG and CDI-WS (Fenson et al., 1993; Marchman et al., 2023) developed by ALSPAC, consisting of 133 and 123 words, respectively. In addition, morphology, word form production and the use of word combinations were assessed at 24 and 38 months of age using sections from the CDI:WS (Fenson et al., 1993; Marchman et al., 2023). Detailed information on each measure is provided below.

*Expressive vocabulary size*

Expressive vocabulary size was assessed at 15 months using an abbreviated version (133 words) of the CDI-WG vocabulary checklist (Fenson et al., 1993; Marchman et al., 2023). For each word, parents indicated whether the child “understands and says” it, “understands only”, or does “neither”. The expressive vocabulary score reflects the number of words for which a parent indicated that a child could “understands and says”.

At 24 and 38 months of age expressive vocabulary size was assessed using an abbreviated wordlist (123 words) from the CDI-WS (Fenson et al., 1993; Marchman et al., 2023). Parents indicated for each word if their child “says” and/or “understands”. Parents could choose one, both, or none of these two options. The total number of words a child could “say”, irrespective of whether the child could “understand”, was recorded as a child’s expressive vocabulary size.

*Receptive vocabulary size*

At 15 months of age receptive vocabulary size was assessed with an abbreviated version (133 words) of the CDI:WG described above. The receptive vocabulary score reflects the number of words for which the parent indicated “understands only”.

At 24 and 38 months, receptive vocabulary was assessed with an abbreviated wordlist from the MacArthur CDI:WS (CDI-WS, 123 words), as described above. The receptive vocabulary score reflects the number of words a child could “understand”, irrespective of whether the child “says” the word. The receptive vocabulary score at 24 months was excluded due to discrepancies in the applied coding scheme (excluding words for which both “says” and “understands” were ticked), and adjusted scores have not yet been released by ALSPAC.

*Morphology*

At 24 and 38 months a score was constructed reflecting the use of common English morphosyntactic rules utilising the four questions of the Word Endings/Part 1 (II.1) section of the ‘Sentences and Grammar’ part of the CDI-WS (Fenson et al., 1993; Marchman et al., 2023). These items assess the use of plurals, the use of possessives, adding ‘ing’ to verbs, and adding ‘ed’ to verbs, reflecting the regular forms for the four morphemes. For each item a score of zero was given if the child did not yet do this and a score of one if a child applied the morphosyntactic rule sometimes or often. Finally, a morphology score was created for each child by summing across the four items.

*Word form production*

As part of the ‘Word Form section’ (II.B) of the CDI-WS (Fenson et al., 1993; Marchman et al., 2023) parents were asked whether or not their child produced 25 specific word forms at 24 and 38 months of age. These 25 items consisted of five plural words and twenty words indicating that something happened in the past, reflecting common irregular forms in both categories. Similar to the expressive vocabulary coding scheme, a score of one was awarded if a parent indicated that a child could “say” a word, irrespective of whether the child could “understand” the word. A score of zero was awarded if a parent indicated that a child “understands” only, or “neither says or understands” a word. Scores were summed across all 25 items to create a measure of word form production.

*Combines words*

At 24 and 38 months of age parents were asked whether their child has begun to combine words as part of the CDI-WS (Fenson et al., 1993). If a parent indicated that their child had not yet begun to do this, a score of zero was awarded. If a parent indicated that their child used word combinations sometimes or often, a score of one was awarded.

## **Appendix S4: Description of psychological instruments**

Motor, personal-social, self-care/symbolic and language development during infancy and toddlerhood were assessed based on abbreviated versions of the Denver Developmental Screening Test (DDST)(Frankenburg & Dodds, 1967), the MacArthur CDI: Words & Gestures (CDI-WG), and the MacArthur CDI: Words & Sentences (CDI-WS)(Fenson et al., 1993; Marchman et al., 2023).

The DDST has largely congruent classifications (co-negativity=0.92 and co-positivity=0.68) with the revised Bayley Infant Mental and Motor Scales and the Stanford-Binet Form LM (Frankenburg, Goldstein, & Camp, 1971). Furthermore, test re-test reliability of the same children seven days apart yielded 97% agreeable scores.

The CDI is also an extensively validated instrument, especially for expressive vocabulary size and grammar (Bleses et al., 2008; Dale, 1991; Law & Roy, 2008; Marchman et al., 2023; van Noort-van der Spek, Franken, Swarte, & Weisglas-Kuperus, 2021; Ring & Fenson, 2000). The correlation between parental CDI assessment and child task performance (20-30 months of age) for word comprehension and production is moderate to high (r=0.55 and r=0.67, respectively)(Ring & Fenson, 2000). CDI-based and laboratory-assessed expressive vocabulary and syntactic development also highly correlate in 24-month old children (r=0.73-0.79)(Dale, 1991). Parental assessments of receptive vocabulary in children below the age of two is more difficult, with reports of low validity, over- and/or underestimation (van Eldik, Schlichting, Iutje Spelberg, van der Meulen, & van der Meulen, 2004; Houston-Price, Mather, & Sakkalou, 2007; Marchman et al., 2023). Longitudinal stability of CDI vocabulary scores furthermore suggests reliability (Dionne, Dale, Boivin, & Plomin, 2003; Frank, Braginsky, Yurovsky, & Marchman, 2021; Verhoef, Shapland, Fisher, Dale, & St Pourcain, 2021).

Within ALSPAC, an abbreviated version of the CDI-WS was applied at 24 and 38 months of age, despite the instrument being developed for children up to 30 months of age. Assessments at 38 months of age might thus be affected by ceiling effects. However, we showed in previous research that the 38-month vocabulary measurements were strongly genetically correlated with scores assessed at 24 months (r_g_=0.74-0.85)(Verhoef et al., 2021), suggesting content validity. In addition, we previously showed that CDI-WS measures at 38 months captured individual variation related to later-life language and literacy abilities (that were assessed using widely applied standardised instruments)(Verhoef, Shapland, Fisher, Dale, & St Pourcain, 2020). Finally, validity and reliability of abbreviated forms, as applied in ALSPAC (see above) is known to be high for tests containing 50-100 words (Marchman et al., 2023).

## **Appendix S5: Data transformation for continuous scores**

To increase statistical power and control type I error while studying genotype-measure associations using linear regression or multivariate variance decompositions, continuous scores were adjusted for covariates and transformed using a fully-adjusted two-stage transformation (Sofer et al., 2019). This procedure included (1) a linear regression of the score on the covariates age, sex, age^2^, age:sex and age^2^:sex, as well as the first ten ancestry-informative genetic principal components (Price et al., 2006), (2) rank-transformation of the residualised scores obtained in (1), and (3) a linear regression of the residualised scores obtained in (2) on the same covariates as in (1).

## **Appendix S6: Construction of language factor scores - methods**

*Factor structure identification*

Following a split-half design, the ALSPAC data was first randomly split into two independent halves (N_half1_=3,437; N_half2_=3,439) that were matched on age, sex and missingness (R:dplyr library, version 1.0.7). In the first half of the sample, the number of factors underlying the ten language measures was identified using eigenvalue decomposition of the Pearson phenotypic correlation matrix. The number of factors to retain was defined following the Kaiser criterion (Kaiser, 1960) and the optimal coordinate criterion (Ruscio & Roche, 2012). Next, exploratory factor analyses (EFA) were conducted within the first half of the sample, estimating loadings of each measure on each factor (factor loading, λ) using a maximum likelihood and oblimin rotation (R:psych library, version 2.2.9). Subsequently, we performed confirmatory factor analysis (CFA) in the second half of the sample, retaining EFA factor loadings (λ) >|0.10|(Gorsuch, 1983) only, and fixing the variance of latent variables to one (known as standardised parametrisation (Devlieger, Mayer, & Rosseel, 2016)). CFA were carried out using oblimin rotation and maximum likelihood allowing for missing data (R:lavaan library, version 0.6-12 (Rosseel, 2012)). The CFA model fit was evaluated based on the comparative fit index (CFI)(Bentler, 1990), the Tucker–Lewis index (TLI)(Tucker & Lewis, 1973), the root mean square error of approximation (RMSEA)(Steiger, 1990) and Standardized Root Mean Squared Residual (SRMR)(Bentler, 1995). The CFI and TLI are incremental fit indices, with values >0.95 indicating relatively good fit (L. Hu & Bentler, 1999) and values ≥0.90 indicating acceptable fit (Kaplan, 2008). RMSEA, on the other hand, is an absolute fit index that assessed how far a hypothesized model is from a perfect model, with a value <0.05 indicating “close fit” and <0.08 indicating reasonable fit (L. Hu & Bentler, 1999). SRMR is an index of approximate model fit with values ≤0.10 indicating acceptable fit (L.-T. Hu & Bentler, 1995). Finally, if CFA model fit was acceptable or good, the same structure was fitted to the full sample to increase statistical power.

*Alternative model specification*

We assessed potential bias in language factor score estimations due to standardised parametrisation (Devlieger et al., 2016) by fixing the highest factor loadings of each latent language factor to one in the full sample (known as unstandardised parametrisation), and proceeded as described above as part of sensitivity analyses.

## **Appendix S7: Genome-based Restricted Maximum-Likelihood analyses**

SNP-heritability and genetic correlation were estimated using genome-based restricted maximum-likelihood (GREML) analyses (Lee, Yang, Goddard, Visscher, & Wray, 2012; Yang, Lee, Goddard, & Visscher, 2011) as implemented in Genome-wide Complex Trait Analysis (GCTA, v1.26.0) software. This method examines each pair of unrelated individuals present in the sample and predicts their measurement score similarity based on their genetic similarity. The latter is captured by a genetic-relationship-matrix (GRM), which was created with PLINK (v1.9)(Purcell et al., 2007) and reflects the genetic relatedness between each pair of individuals. The GRM was restricted to individuals with a genetic relationship <0.05 and was derived based on directly genotyped SNPs only.

## **Appendix S8: The Cholesky decomposition as a multivariate form of a Direction of Causation model**

The Direction of Causation (DoC) model is a technique applied in twin research studying genetic covariance (Verhulst & Estabrook, 2012). In short, the DoC model predicts different cross-twin cross-trait correlations depending on which direction causation flows, similar to cross-lagged panel studies or other longitudinal methods (Verhulst & Estabrook, 2012). The Cholesky Decomposition is a multivariate form of a DoC model, and is, here, applied to model variation in a sample of unrelated children.

## **Appendix S9: Evaluating consistency with developmental relationships**

According to Cheverud’s conjecture, genetic and environmental effects are thought to act on growth and development in a similar manner (Cheverud, 1988). Indeed, phenotypic correlations are found to be largely predictive of genetic correlations in humans (Sodini, Kemper, Wray, & Trzaskowski, 2018). Consequently, in the presence of genuine developmental relationships between measures, genetic and residual association patterns are thought to converge (Clapp Sullivan et al., 2024; Shapland et al., 2021). Though solely converging genetic and residual effects do not prove causal relationships, they can be interpreted as evidence supporting the existence of developmental links. In case of evidence for residual links only, this could represent measurement bias and/or error. For example, measurements could be linked because they were all assessed by the same parent.

## **Appendix S10: Construction of language factor scores - results**

We reduced the number of language measures concurrently studied with developmental genetic models by identifying overarching language factors and estimating representative language factor scores. Exploratory factor analysis using phenotypic correlations estimated in the first half of the sample revealed three underlying interrelated factors (Figure S3-S4). Confirmatory factor analysis in the second half of the sample following standardised parametrisation (i.e. fixing the variance of latent factors to one) showed that the identified model structure had good fit (Figure S5).

# **Supplementary Tables**

## **Table S1: Phenotypic correlations between transformed measures**

| **Measure 1** | **Measure 2** | **r_p_** | **95%-CI low** | **95%-CI up** |
| --- | --- | --- | --- | --- |
| Fine motor 6M (DDST) | Gross motor 6M (DDST) | 0.41 | 0.39 | 0.43 |
|  | Personal social 6M (DDST) | 0.44 | 0.42 | 0.46 |
|  | Self-care/symbolic 15M (CDI-WG) | 0.28 | 0.26 | 0.31 |
|  | Expressive vocabulary 15M (CDI-WG) | 0.24 | 0.22 | 0.27 |
|  | Expressive vocabulary 24M (CDI-WS) | 0.22 | 0.20 | 0.25 |
|  | Expressive vocabulary 38M (CDI-WS) | 0.16 | 0.14 | 0.19 |
|  | Morphology 24M (CDI-WS) | 0.21 | 0.19 | 0.24 |
|  | Morphology 38M (CDI-WS) | 0.10 | 0.08 | 0.13 |
|  | Receptive vocabulary 15M (CDI-WG) | 0.28 | 0.25 | 0.30 |
|  | Receptive vocabulary 38M (CDI-WS) | 0.14 | 0.12 | 0.17 |
|  | Word form production 38M (CDI-WS) | 0.18 | 0.15 | 0.20 |
|  | Combines words 24M (CDI-WS) | 0.13 | 0.10 | 0.15 |
|  | Combines words 38M (CDI-WS) | 0.05 | 0.02 | 0.08 |
| Gross motor 6M (DDST) | Personal social 6M (DDST) | 0.33 | 0.31 | 0.35 |
|  | Self-care/symbolic 15M (CDI-WG) | 0.24 | 0.22 | 0.26 |
|  | Expressive vocabulary 15M (CDI-WG) | 0.21 | 0.19 | 0.24 |
|  | Expressive vocabulary 24M (CDI-WS) | 0.18 | 0.15 | 0.20 |
|  | Expressive vocabulary 38M (CDI-WS) | 0.12 | 0.10 | 0.15 |
|  | Morphology 24M (CDI-WS) | 0.15 | 0.13 | 0.18 |
|  | Morphology 38M (CDI-WS) | 0.09 | 0.06 | 0.12 |
|  | Receptive vocabulary 15M (CDI-WG) | 0.23 | 0.20 | 0.25 |
|  | Receptive vocabulary 38M (CDI-WS) | 0.11 | 0.08 | 0.14 |
|  | Word form production 38M (CDI-WS) | 0.11 | 0.09 | 0.14 |
|  | Combines words 24M (CDI-WS) | 0.12 | 0.09 | 0.15 |
|  | Combines words 38M (CDI-WS) | 0.04 | 0.01 | 0.07 |
| Personal social 6M (DDST) | Self-care/symbolic 15M (CDI-WG) | 0.29 | 0.26 | 0.31 |
|  | Expressive vocabulary 15M (CDI-WG) | 0.22 | 0.19 | 0.24 |
|  | Expressive vocabulary 24M (CDI-WS) | 0.18 | 0.15 | 0.20 |
|  | Expressive vocabulary 38M (CDI-WS) | 0.13 | 0.11 | 0.16 |
|  | Morphology 24M (CDI-WS) | 0.16 | 0.13 | 0.18 |
|  | Morphology 38M (CDI-WS) | 0.09 | 0.06 | 0.11 |
|  | Receptive vocabulary 15M (CDI-WG) | 0.28 | 0.25 | 0.30 |
|  | Receptive vocabulary 38M (CDI-WS) | 0.14 | 0.11 | 0.16 |
|  | Word form production 38M (CDI-WS) | 0.14 | 0.11 | 0.17 |
|  | Combines words 24M (CDI-WS) | 0.12 | 0.09 | 0.14 |
|  | Combines words 38M (CDI-WS) | 0.05 | 0.03 | 0.08 |
| Self-care/ symbolic 15M (CDI-WG) | Expressive vocabulary 15M (CDI-WG) | 0.38 | 0.35 | 0.40 |
|  | Expressive vocabulary 24M (CDI-WS) | 0.37 | 0.35 | 0.39 |
|  | Expressive vocabulary 38M (CDI-WS) | 0.27 | 0.24 | 0.29 |
|  | Morphology 24M (CDI-WS) | 0.28 | 0.26 | 0.31 |
|  | Morphology 38M (CDI-WS) | 0.15 | 0.12 | 0.18 |
|  | Receptive vocabulary 15M (CDI-WG) | 0.51 | 0.49 | 0.52 |
|  | Receptive vocabulary 38M (CDI-WS) | 0.24 | 0.21 | 0.26 |
|  | Word form production 38M (CDI-WS) | 0.22 | 0.20 | 0.25 |
|  | Combines words 24M (CDI-WS) | 0.21 | 0.18 | 0.23 |
|  | Combines words 38M (CDI-WS) | 0.08 | 0.05 | 0.11 |
| Expressive vocabulary 15M (CDI-WG) | Expressive vocabulary 24M (CDI-WS) | 0.53 | 0.51 | 0.55 |
|  | Expressive vocabulary 38M (CDI-WS) | 0.24 | 0.21 | 0.26 |
|  | Morphology 24M (CDI-WS) | 0.44 | 0.42 | 0.47 |
|  | Morphology 38M (CDI-WS) | 0.16 | 0.13 | 0.18 |
|  | Receptive vocabulary 15M (CDI-WG) | 0.53 | 0.51 | 0.55 |
|  | Receptive vocabulary 38M (CDI-WS) | 0.20 | 0.17 | 0.22 |
|  | Word form production 38M (CDI-WS) | 0.21 | 0.19 | 0.24 |
|  | Combines words 24M (CDI-WS) | 0.30 | 0.28 | 0.33 |
|  | Combines words 38M (CDI-WS) | 0.06 | 0.04 | 0.09 |
| Expressive vocabulary 24M (CDI-WS) | Expressive vocabulary 38M (CDI-WS) | 0.49 | 0.47 | 0.51 |
|  | Morphology 24M (CDI-WS) | 0.73 | 0.71 | 0.74 |
|  | Morphology 38M (CDI-WS) | 0.30 | 0.27 | 0.32 |
|  | Receptive vocabulary 15M (CDI-WG) | 0.45 | 0.42 | 0.47 |
|  | Receptive vocabulary 38M (CDI-WS) | 0.39 | 0.37 | 0.41 |
|  | Word form production 38M (CDI-WS) | 0.42 | 0.40 | 0.45 |
|  | Combines words 24M (CDI-WS) | 0.53 | 0.51 | 0.54 |
|  | Combines words 38M (CDI-WS) | 0.15 | 0.12 | 0.17 |
| Expressive vocabulary 38M (CDI-WS) | Morphology 24M (CDI-WS) | 0.37 | 0.35 | 0.39 |
|  | Morphology 38M (CDI-WS) | 0.42 | 0.40 | 0.44 |
|  | Receptive vocabulary 15M (CDI-WG) | 0.32 | 0.29 | 0.34 |
|  | Receptive vocabulary 38M (CDI-WS) | 0.61 | 0.60 | 0.63 |
|  | Word form production 38M (CDI-WS) | 0.65 | 0.64 | 0.67 |
|  | Combines words 24M (CDI-WS) | 0.29 | 0.26 | 0.31 |
|  | Combines words 38M (CDI-WS) | 0.20 | 0.18 | 0.23 |
| Morphology 24M (CDI-WS) | Morphology 38M (CDI-WS) | 0.29 | 0.27 | 0.32 |
|  | Receptive vocabulary 15M (CDI-WG) | 0.35 | 0.33 | 0.37 |
|  | Receptive vocabulary 38M (CDI-WS) | 0.29 | 0.26 | 0.31 |
|  | Word form production 38M (CDI-WS) | 0.37 | 0.34 | 0.39 |
|  | Combines words 24M (CDI-WS) | 0.48 | 0.46 | 0.50 |
|  | Combines words 38M (CDI-WS) | 0.11 | 0.08 | 0.14 |
| Morphology 38M (CDI-WS) | Receptive vocabulary 15M (CDI-WG) | 0.17 | 0.15 | 0.20 |
|  | Receptive vocabulary 38M (CDI-WS) | 0.26 | 0.24 | 0.29 |
|  | Word form production 38M (CDI-WS) | 0.39 | 0.36 | 0.41 |
|  | Combines words 24M (CDI-WS) | 0.30 | 0.28 | 0.32 |
|  | Combines words 38M (CDI-WS) | 0.27 | 0.25 | 0.29 |
| Receptive vocabulary 15M (CDI-WG) | Receptive vocabulary 38M (CDI-WS) | 0.27 | 0.25 | 0.30 |
|  | Word form production 38M (CDI-WS) | 0.28 | 0.25 | 0.30 |
|  | Combines words 24M (CDI-WS) | 0.18 | 0.16 | 0.21 |
|  | Combines words 38M (CDI-WS) | 0.05 | 0.03 | 0.08 |
| Receptive vocabulary 38M (CDI-WS) | Word form production 38M (CDI-WS) | 0.42 | 0.40 | 0.44 |
|  | Combines words 24M (CDI-WS) | 0.23 | 0.20 | 0.25 |
|  | Combines words 38M (CDI-WS) | 0.10 | 0.08 | 0.13 |
| Word form production 38M (CDI-WS) | Combines words 24M (CDI-WS) | 0.25 | 0.22 | 0.27 |
|  | Combines words 38M (CDI-WS) | 0.18 | 0.15 | 0.20 |
| Combines words 24M (CDI-WS) | Combines words 38M (CDI-WS) | 0.19 | 0.17 | 0.22 |

Correlations among predictor and outcome measures at the behavioural level (r_p_) with corresponding 95%-confidence intervals. Estimates were obtained for transformed scores using Pearson correlation in R (R:dplyr library, v.1.1.4).

Abbreviations: CDI-WG, Communicative Development Inventories – Words and Gestures; CDI-WS; Communicative Development Inventories – Words and Sentences; DDST, Denver Developmental Screening Test; M, months; 95%-CI, 95% confidence interval

## **Table S2: Phenotypic correlations between untransformed measures**

| **Measure 1** | **Measure 2** | **r_p_** | **95%-CI low** | **95%-CI up** |
| --- | --- | --- | --- | --- |
| Fine motor 6M (DDST) | Gross motor 6M (DDST) | 0.48 | 0.46 | 0.50 |
|  | Personal social 6M (DDST) | 0.48 | 0.46 | 0.50 |
|  | Self-care/symbolic 15M (CDI-WG) | 0.29 | 0.27 | 0.32 |
|  | Expressive vocabulary 15M (CDI-WG) | 0.25 | 0.23 | 0.28 |
|  | Expressive vocabulary 24M (CDI-WS) | 0.24 | 0.22 | 0.27 |
|  | Expressive vocabulary 38M (CDI-WS) | 0.18 | 0.15 | 0.20 |
|  | Morphology 24M (CDI-WS) | 0.24 | 0.21 | 0.26 |
|  | Morphology 38M (CDI-WS) | 0.12 | 0.10 | 0.15 |
|  | Receptive vocabulary 15M (CDI-WG) | 0.28 | 0.26 | 0.31 |
|  | Receptive vocabulary 38M (CDI-WS) | 0.16 | 0.14 | 0.19 |
|  | Word form production 38M (CDI-WS) | 0.19 | 0.17 | 0.22 |
|  | Combines words 24M (CDI-WS) | 0.13 | 0.11 | 0.16 |
|  | Combines words 38M (CDI-WS) | 0.05 | 0.03 | 0.08 |
| Gross motor 6M (DDST) | Personal social 6M (DDST) | 0.38 | 0.36 | 0.40 |
|  | Self-care/symbolic 15M (CDI-WG) | 0.24 | 0.21 | 0.26 |
|  | Expressive vocabulary 15M (CDI-WG) | 0.21 | 0.18 | 0.23 |
|  | Expressive vocabulary 24M (CDI-WS) | 0.18 | 0.16 | 0.21 |
|  | Expressive vocabulary 38M (CDI-WS) | 0.12 | 0.09 | 0.15 |
|  | Morphology 24M (CDI-WS) | 0.16 | 0.14 | 0.19 |
|  | Morphology 38M (CDI-WS) | 0.09 | 0.07 | 0.12 |
|  | Receptive vocabulary 15M (CDI-WG) | 0.22 | 0.20 | 0.25 |
|  | Receptive vocabulary 38M (CDI-WS) | 0.12 | 0.09 | 0.14 |
|  | Word form production 38M (CDI-WS) | 0.12 | 0.10 | 0.15 |
|  | Combines words 24M (CDI-WS) | 0.12 | 0.10 | 0.15 |
|  | Combines words 38M (CDI-WS) | 0.04 | 0.01 | 0.07 |
| Personal social 6M (DDST) | Self-care/symbolic 15M (CDI-WG) | 0.28 | 0.26 | 0.31 |
|  | Expressive vocabulary 15M (CDI-WG) | 0.23 | 0.20 | 0.25 |
|  | Expressive vocabulary 24M (CDI-WS) | 0.18 | 0.16 | 0.21 |
|  | Expressive vocabulary 38M (CDI-WS) | 0.14 | 0.11 | 0.17 |
|  | Morphology 24M (CDI-WS) | 0.17 | 0.15 | 0.20 |
|  | Morphology 38M (CDI-WS) | 0.10 | 0.07 | 0.12 |
|  | Receptive vocabulary 15M (CDI-WG) | 0.28 | 0.26 | 0.31 |
|  | Receptive vocabulary 38M (CDI-WS) | 0.15 | 0.13 | 0.18 |
|  | Word form production 38M (CDI-WS) | 0.15 | 0.12 | 0.18 |
|  | Combines words 24M (CDI-WS) | 0.12 | 0.09 | 0.15 |
|  | Combines words 38M (CDI-WS) | 0.05 | 0.02 | 0.08 |
| Self-care/ symbolic 15M (CDI-WG) | Expressive vocabulary 15M (CDI-WG) | 0.42 | 0.40 | 0.44 |
|  | Expressive vocabulary 24M (CDI-WS) | 0.38 | 0.36 | 0.40 |
|  | Expressive vocabulary 38M (CDI-WS) | 0.29 | 0.26 | 0.31 |
|  | Morphology 24M (CDI-WS) | 0.32 | 0.29 | 0.34 |
|  | Morphology 38M (CDI-WS) | 0.16 | 0.14 | 0.19 |
|  | Receptive vocabulary 15M (CDI-WG) | 0.52 | 0.50 | 0.54 |
|  | Receptive vocabulary 38M (CDI-WS) | 0.26 | 0.23 | 0.28 |
|  | Word form production 38M (CDI-WS) | 0.23 | 0.20 | 0.25 |
|  | Combines words 24M (CDI-WS) | 0.21 | 0.19 | 0.24 |
|  | Combines words 38M (CDI-WS) | 0.08 | 0.05 | 0.10 |
| Expressive vocabulary 15M (CDI-WG) | Expressive vocabulary 24M (CDI-WS) | 0.56 | 0.54 | 0.58 |
|  | Expressive vocabulary 38M (CDI-WS) | 0.27 | 0.24 | 0.29 |
|  | Morphology 24M (CDI-WS) | 0.49 | 0.47 | 0.51 |
|  | Morphology 38M (CDI-WS) | 0.20 | 0.18 | 0.23 |
|  | Receptive vocabulary 15M (CDI-WG) | 0.55 | 0.54 | 0.57 |
|  | Receptive vocabulary 38M (CDI-WS) | 0.22 | 0.20 | 0.24 |
|  | Word form production 38M (CDI-WS) | 0.24 | 0.21 | 0.26 |
|  | Combines words 24M (CDI-WS) | 0.35 | 0.33 | 0.37 |
|  | Combines words 38M (CDI-WS) | 0.08 | 0.05 | 0.10 |
| Expressive vocabulary 24M (CDI-WS) | Expressive vocabulary 38M (CDI-WS) | 0.51 | 0.49 | 0.52 |
|  | Morphology 24M (CDI-WS) | 0.76 | 0.75 | 0.77 |
|  | Morphology 38M (CDI-WS) | 0.33 | 0.31 | 0.35 |
|  | Receptive vocabulary 15M (CDI-WG) | 0.46 | 0.44 | 0.48 |
|  | Receptive vocabulary 38M (CDI-WS) | 0.42 | 0.40 | 0.44 |
|  | Word form production 38M (CDI-WS) | 0.42 | 0.40 | 0.44 |
|  | Combines words 24M (CDI-WS) | 0.52 | 0.50 | 0.54 |
|  | Combines words 38M (CDI-WS) | 0.14 | 0.11 | 0.16 |
| Expressive vocabulary 38M (CDI-WS) | Morphology 24M (CDI-WS) | 0.39 | 0.37 | 0.41 |
|  | Morphology 38M (CDI-WS) | 0.35 | 0.33 | 0.37 |
|  | Receptive vocabulary 15M (CDI-WG) | 0.34 | 0.31 | 0.36 |
|  | Receptive vocabulary 38M (CDI-WS) | 0.65 | 0.63 | 0.66 |
|  | Word form production 38M (CDI-WS) | 0.64 | 0.63 | 0.65 |
|  | Combines words 24M (CDI-WS) | 0.26 | 0.24 | 0.29 |
|  | Combines words 38M (CDI-WS) | 0.14 | 0.12 | 0.16 |
| Morphology 24M (CDI-WS) | Morphology 38M (CDI-WS) | 0.33 | 0.31 | 0.35 |
|  | Receptive vocabulary 15M (CDI-WG) | 0.38 | 0.36 | 0.40 |
|  | Receptive vocabulary 38M (CDI-WS) | 0.33 | 0.30 | 0.35 |
|  | Word form production 38M (CDI-WS) | 0.37 | 0.35 | 0.40 |
|  | Combines words 24M (CDI-WS) | 0.49 | 0.47 | 0.51 |
|  | Combines words 38M (CDI-WS) | 0.11 | 0.08 | 0.14 |
| Morphology 38M (CDI-WS) | Receptive vocabulary 15M (CDI-WG) | 0.19 | 0.16 | 0.21 |
|  | Receptive vocabulary 38M (CDI-WS) | 0.28 | 0.26 | 0.31 |
|  | Word form production 38M (CDI-WS) | 0.36 | 0.34 | 0.39 |
|  | Combines words 24M (CDI-WS) | 0.32 | 0.29 | 0.34 |
|  | Combines words 38M (CDI-WS) | 0.25 | 0.22 | 0.27 |
| Receptive vocabulary 15M (CDI-WG) | Receptive vocabulary 38M (CDI-WS) | 0.29 | 0.26 | 0.31 |
|  | Word form production 38M (CDI-WS) | 0.29 | 0.26 | 0.31 |
|  | Combines words 24M (CDI-WS) | 0.20 | 0.17 | 0.22 |
|  | Combines words 38M (CDI-WS) | 0.06 | 0.03 | 0.08 |
| Receptive vocabulary 38M (CDI-WS) | Word form production 38M (CDI-WS) | 0.45 | 0.42 | 0.46 |
|  | Combines words 24M (CDI-WS) | 0.23 | 0.21 | 0.26 |
|  | Combines words 38M (CDI-WS) | 0.10 | 0.08 | 0.13 |
| Word form production 38M (CDI-WS) | Combines words 24M (CDI-WS) | 0.24 | 0.21 | 0.26 |
|  | Combines words 38M (CDI-WS) | 0.14 | 0.11 | 0.16 |
| Combines words 24M (CDI-WS) | Combines words 38M (CDI-WS) | 0.20 | 0.17 | 0.22 |

Correlations among predictor and outcome measures at the behavioural level (r_p_) with corresponding 95%-confidence intervals. Estimates were obtained for untransformed scores using Spearman correlation in R if one of the variables was continuous, and using Pearson correlation if both variables were binary (R:dplyr library, v.1.1.4).

Abbreviations: CDI-WG, Communicative Development Inventories – Words and Gestures; CDI-WS; Communicative Development Inventories – Words and Sentences; DDST, Denver Developmental Screening Test; M, months; 95%-CI, 95% confidence interval

## **Table S3: Developmental genetic relationships of gross motor behaviour (6 months) and self-care/symbolic actions (15 months) with language factor scores**

| **Factor** | **Trait** | **λ(SE)** | ***P*** | **var(SE)** |
| --- | --- | --- | --- | --- |
| A1 | Gross motor 6M | 0.41(0.07) | 8x10^-10^ | 0.17(0.06) |
|  | Self-care/symbolic 15M | 0.22(0.09) | 0.02 | 0.05(0.04) |
|  | Language FS_15M_ | 0.15(0.09) | 0.11 | 0.02(0.03) |
|  | Language FS_24M_ | 0.17(0.09) | 0.07 | 0.03(0.03) |
|  | Language FS_38M_ | 0.13(0.09) | 0.16 | 0.02(0.03) |
| A2 | Self-care/symbolic 15M | 0.38(0.07) | 4x10^-7^ | 0.14(0.06) |
|  | Language FS_15M_ | 0.26(0.09) | 4x10^-3^ | 0.07(0.05) |
|  | Language FS_24M_ | 0.28(0.10) | 4x10^-3^ | 0.08(0.05) |
|  | Language FS_38M_ | 0.30(0.10) | 2x10^-3^ | 0.09(0.06) |
| A3 | Language FS_15M_ | 0.19(0.10) | 0.07 | 0.04(0.04) |
|  | Language FS_24M_ | 0.16(0.15) | 0.28 | 0.03(0.05) |
|  | Language FS_38M_ | 0.10(0.17) | 0.54 | 0.01(0.04) |
| A4 | Language FS_24M_ | 0.28(0.07) | 3x10^-5^ | 0.08(0.04) |
|  | Language FS_38M_ | 0.14(0.10) | 0.16 | 0.02(0.03) |
| A5 | Language FS_38M_ | 0.16(0.11) | 0.14 | 0.03(0.04) |
| E1 | Gross motor 6M | 0.91(0.03) | <1x10^-10^ | 0.83(0.06) |
|  | Self-care/symbolic 15M | 0.17(0.04) | 1x10^-4^ | 0.03(0.01) |
|  | Language FS_15M_ | 0.21(0.04) | 8x10^-7^ | 0.04(0.02) |
|  | Language FS_24M_ | 0.15(0.04) | 8x10^-4^ | 0.02(0.01) |
|  | Language FS_38M_ | 0.10(0.04) | 0.02 | 0.01(0.01) |
| E2 | Self-care/symbolic 15M | 0.88(0.03) | <1x10^-10^ | 0.78(0.05) |
|  | Language FS_15M_ | 0.41(0.04) | <1x10^-10^ | 0.17(0.03) |
|  | Language FS_24M_ | 0.25(0.04) | 3x10^-9^ | 0.06(0.02) |
|  | Language FS_38M_ | 0.17(0.04) | 4x10^-5^ | 0.03(0.01) |
| E3 | Language FS_15M_ | 0.81(0.02) | <1x10^-10^ | 0.66(0.04) |
|  | Language FS_24M_ | 0.45(0.04) | <1x10^-10^ | 0.20(0.03) |
|  | Language FS_38M_ | 0.35(0.04) | <1x10^-10^ | 0.12(0.03) |
| E4 | Language FS_24M_ | 0.71(0.02) | <1x10^-10^ | 0.50(0.04) |
|  | Language FS_38M_ | 0.37(0.04) | <1x10^-10^ | 0.13(0.03) |
| E5 | Language FS_38M_ | 0.74(0.02) | <1x10^-10^ | 0.54(0.03) |

Cholesky decomposition of gross motor skills (6 months), self-care/symbolic actions (15 months), and the three estimated language factor scores (Language FS_15m_, Language FS_24m_ and Language FS_38m_), in that order. The phenotypic covariance of the five measures was dissected into five genomic (A1-A5) and five non-genomic/residual factors (E1-E5). Analyses were based on all available observations for children across development (N≤6,975) and estimated with Genetic-relationship matrix structural equation modelling (grmsem, v.1.1.2) (Figure 2). Path coefficients (λ) and the corresponding variance explained (var) are shown with their respective standard errors in brackets. Individual-level data were retrieved from the Avon Longitudinal Study of Parents and Children.

Abbreviations: FS, factor score; M, months; SE, standard error; var, variance

## **Table S4: Developmental genetic relationships of gross motor behaviour (6 months) and self-care/symbolic actions (15 months) with language measures (15 months)**

| **Factor** | **Trait** | **λ(SE)** | ***P*** | **var(SE)** |
| --- | --- | --- | --- | --- |
| A1 | Gross motor 6M | 0.41(0.07) | 9x10^-10^ | 0.17(0.06) |
|  | Self-care/symbolic 15M | 0.22(0.09) | 0.02 | 0.05(0.04) |
|  | Exp voc 15M | 0.10(0.09) | 0.29 | 0.01(0.02) |
|  | Rec voc 15M | 0.12(0.09) | 0.19 | 0.01(0.02) |
| A2 | Self-care/symbolic 15M | 0.37(0.08) | 1x10^-6^ | 0.14(0.06) |
|  | Exp voc 15M | 0.18 (0.10) | 0.06 | 0.03(0.04) |
|  | Rec voc 15M | 0.19(0.09) | 0.05 | 0.03(0.04) |
| A3 | Exp voc 15M | 0.26(0.09) | 3x10^-3^ | 0.07(0.05) |
|  | Rec voc 15M | 0.09(0.11) | 0.42 | 0.01(0.02) |
| A4 | Rec voc 15M | 0.12(0.13) | 0.35 | 0.01(0.03) |
| E1 | Gross motor 6M | 0.91(0.03) | <1x10^-10^ | 0.83(0.06) |
|  | Self-care/symbolic 15M | 0.17(0.04) | 1x10^-4^ | 0.03(0.01) |
|  | Exp voc 15M | 0.19(0.09) | 7x10^-6^ | 0.04(0.02) |
|  | Rec voc 15M | 0.20(0.04) | 2x10^-6^ | 0.02(0.01) |
| E2 | Self-care/symbolic 15M | 0.89(0.03) | <1x10^-10^ | 0.78(0.05) |
|  | Exp voc 15M | 0.29(0.04) | <1x10^-10^ | 0.17(0.03) |
|  | Rec voc 15M | 0.43(0.04) | <1x10^-10^ | 0.06(0.02) |
| E3 | Exp voc 15M | 0.88(0.03) | <1x10^-10^ | 0.66(0.04) |
|  | Rec voc 15M | 0.34(0.03) | <1x10^-10^ | 0.20(0.03) |
| E4 | Rec voc 15M | 0.77(0.02) | <1x10^-10^ | 0.50(0.04) |

Cholesky decomposition of gross motor skills (6 months), self-care/symbolic actions (15 months), expressive and receptive vocabulary size (both at 15 months), in that order. The covariance of the five measures was dissected into four genomic (A1-A4) and four non-genomic/residual factors (E1-E4). Analyses were based on all available observations for children across development (N≤6,908) and estimated with Genetic-relationship matrix structural equation modelling (grmsem, v.1.1.2) (Figure 3). Path coefficients (λ) and the corresponding variance explained (var) are shown with their respective standard errors in brackets. Individual-level data were retrieved from the Avon Longitudinal Study of Parents and Children.

Abbreviations: Exp, expressive; M, months; rec, receptive; SE, standard error; var, variance; voc, vocabulary

## **Table S5: Developmental genetic relationships of gross motor behaviour (6 months) and self-care/symbolic actions (15 months) with language measures (24 months)**

| **Factor** | **Trait** | **λ(SE)** | ***P*** | **var(SE)** |
| --- | --- | --- | --- | --- |
| A1 | Gross motor 6M | 0.41(0.07) | 1x10^-9^ | 0.17(0.05) |
|  | Self-care/symbolic 15M | 0.22(0.09) | 0.02 | 0.05(0.04) |
|  | Exp voc 24M | 0.16(0.10) | 0.09 | 0.03(0.03) |
|  | Morphology 24M | 0.16(0.10)) | 0.10 | 0.03(0.03) |
|  | Combines words 24M | 0.15(0.10) | 0.12 | 0.02(0.03) |
| A2 | Self-care/symbolic 15M | 0.38(0.07) | 6x10^-8^ | 0.15(0.05) |
|  | Exp voc 24M | 0.25(0.09) | 0.01 | 0.06(0.05) |
|  | Morphology 24M | 0.18(0.10) | 0.08 | 0.03(0.04) |
|  | Combines words 24M | 0.03 (0.10) | 0.79 | 0.001(0.01) |
| A3 | Exp voc 24M | 0.28(0.09) | 2x10^-3^ | 0.08(0.05) |
|  | Morphology 24M | 0.32(0.10) | 2x10^-3^ | 0.11(0.07) |
|  | Combines words 24M | 0.29(0.09) | 2x10^-3^ | 0.08(0.05) |
| A4 | Morphology 24M | 0.21(0.09) | 0.01 | 0.04(0.04) |
|  | Combines words 24M | -0.04(0.12) | 0.73 | 0.002(0.01) |
| A5 | Combines words 24M | -7x10^-6^(0.25) | 1.00 | 4x10^-11^(3x10^-6^) |
| E1 | Gross motor 6M | 0.91(0.03) | <1x10^-10^ | 0.83(0.06) |
|  | Self-care/symbolic 15M | 0.17(0.04) | 6x10^-5^ | 0.03(0.01) |
|  | Exp voc 24M | 0.12(0.04) | 0.01 | 0.04(0.02) |
|  | Morphology 24M | 0.10(0.05) | 0.03 | 0.02(0.01) |
|  | Combines words 24M | 0.07(0.04) | 0.12 | 0.01(0.01) |
| E2 | Self-care/symbolic 15M | 0.88(0.03) | <1x10^-10^ | 0.78(0.05) |
|  | Exp voc 24M | 0.25(0.04) | 5x10^-9^ | 0.17(0.03) |
|  | Morphology 24M | 0.18(0.04) | 3x10^-5^ | 0.06(0.02) |
|  | Combines words 24M | 0.17(0.04) | 5x10^-5^ | 0.03(0.01) |
| E3 | Exp voc 24M | 0.87(0.03) | <1x10^-10^ | 0.66(0.04) |
|  | Morphology 24M | 0.58(0.04) | <1x10^-10^ | 0.20(0.03) |
|  | Combines words 24M | 0.42(0.04) | <1x10^-10^ | 0.12(0.03) |
| E4 | Morphology 24M | 0.64(0.02) | <1x10^-10^ | 0.50(0.04) |
|  | Combines words 24M | 0.13(0.04) | 5x10^-4^ | 0.13(0.03) |
| E5 | Combines words 24M | 0.82(0.02) | <1x10^-10^ | 0.54(0.03) |

Cholesky decomposition of gross motor skills (6 months), self-care/symbolic actions (15 months), expressive vocabulary size (24 months), morphology (24 months) and the use of word combinations (24 months), in that order. The phenotypic covariance of the five measures was dissected into five genomic (A1-A5) and five non-genomic/residual factors (E1-E5). Analyses were based on all available observations for children across development (N≤6,976) and estimated with Genetic-relationship matrix structural equation modelling (grmsem, v.1.1.2) (Figure 4). Path coefficients (λ) and the corresponding variance explained (var) are shown with their respective standard errors in brackets. Individual-level data were retrieved from the Avon Longitudinal Study of Parents and Children.

Abbreviations: Exp, expressive; M, months; rec, receptive; SE, standard error; var, variance; voc, vocabulary

## **Table S6: Developmental genetic relationships of gross motor behaviour (6 months) and self-care/symbolic actions (15 months) with language measures (38 months)**

| **Factor** | **Trait** | **λ(SE)** | ***P*** | **var(SE)** |
| --- | --- | --- | --- | --- |
| A1 | Gross motor 6M | 0.41(0.07) | 4x10^-10^ | 0.17(0.05) |
|  | Self-care/symbolic 15M | 0.22(0.09) | 0.01 | 0.05(0.04) |
|  | Exp voc 38M | 0.14(0.09) | 0.13 | 0.02(0.03) |
|  | Morphology 38M | 0.29(0.10) | 3x10^-3^ | 0.08(0.05) |
|  | Rec voc 38M | 0.09(0.09) | 0.33 | 0.01(0.02) |
|  | Word form prod 38M | 0.21(0.10) | 0.03 | 0.05(0.04) |
|  | Combines words 38M | 0.05(0.09) | 0.54 | 3x10^-3^(0.01) |
| A2 | Self-care/symbolic 15M | 0.40(0.07) | 2x10^-9^ | 0.16(0.05) |
|  | Exp voc 38M | 0.26(0.09) | 4x10^-3^ | 0.07(0.05) |
|  | Morphology 38M | 0.11(0.11) | 0.31 | 0.01(0.02) |
|  | Rec voc 38M | 0.19(0.09) | 0.04 | 0.04(0.04) |
|  | Word form prod 38M | 0.16(0.10) | 0.11 | 0.03(0.03) |
|  | Combines words 38M | 0.21(0.09) | 0.02 | 0.04(0.04) |
| A3 | Exp voc 38M | 0.26(0.10) | 0.01 | 0.07(0.05) |
|  | Morphology 38M | 0.20(0.14) | 0.16 | 0.04(0.06) |
|  | Rec voc 38M | 0.22(0.12) | 0.06 | 0.05(0.05) |
|  | Word form prod 38M | 0.18(0.13) | 0.17 | 0.03(0.05) |
|  | Combines words 38M | 0.003(0.13) | 0.98 | 8x10^-6^(7x10^-4^) |
| A4 | Morphology 38M | 0.11(0.16) | 0.49 | 0.01(0.04) |
|  | Rec voc 38M | 0.10(0.18) | 0.56 | 0.01(0.04) |
|  | Word form prod 38M | 0.11(0.21) | 0.61 | 0.01(0.05) |
|  | Combines words 38M | 0.10(0.24) | 0.67 | 0.01(0.05) |
| A5 | Rec voc 38M | 0.14(0.013) | 0.28 | 0.02(0.04) |
|  | Word form prod 38M | -0.19(0.11) | 0.07 | 0.04(0.04) |
|  | Combines words 38M | 0.22(0.12) | 0.06 | 0.05(0.05) |
| A6 | Word form prod 38M | 4x10^-4^(0.44) | 1.00 | 1x10^-7^(3x10^-4^) |
|  | Combines words 38M | -2x10^-4^(0.27) | 1.00 | 4x10^-8^(1x10^-4^) |
| A7 | Combines words 38M | 2x10^-6^(0.18) | 1.00 | 3x10^-12^(6x10^-7^) |

**Table S6 continued: Developmental genetic relationships of gross motor (6 months) and self-care/symbolic actions (15 months) with language measures (38 months)**

| **Factor** | **Trait** | **λ(SE)** | ***P*** | **var(SE)** |
| --- | --- | --- | --- | --- |
| E1 | Gross motor 6M | 0.91(0.03) | <1x10^-10^ | 0.83(0.05) |
|  | Self-care/symbolic 15M | 0.17(0.04) | 7x10^-5^ | 0.03(0.01) |
|  | Exp voc 38M | 0.07(0.04) | 0.08 | 0.01(0.01) |
|  | Morphology 38M | -0.02(0.04) | 0.57 | 6x10^-4^(2x10^-3^) |
|  | Rec voc 38M | 0.08(0.04) | 0.05 | 0.01(0.01) |
|  | Word form prod 38M | 0.03(0.04) | 0.44 | 1x10^-3^(3x10^-3^) |
|  | Combines words 38M | 0.02(0.04) | 0.57 | 5x10^-4^(2x10^-3^) |
| E2 | Self-care/symbolic 15M | 0.87(0.03) | <1x10^-10^ | 0.76(0.05) |
|  | Exp voc 38M | 0.14(0.04 | 8x10^-4^ | 0.02(0.01) |
|  | Morphology 38M | 0.06(0.04) | 0.21 | 3x10^-3^(5x10^-3^) |
|  | Rec voc 38M | 0.15(0.04) | 4x10^-4^ | 0.02(0.01) |
|  | Word form prod 38M | 0.12(0.04) | 5x10^-3^ | 0.01(0.01) |
|  | Combines words 38M | -0.02(0.04) | 0.64 | 4x10^-4^(2x10^-3^) |
| E3 | Exp voc 38M | 0.91(0.03) | <1x10^-10^ | 0.82(0.05) |
|  | Morphology 38M | 0.33(0.04) | <1x10^-10^ | 0.11(0.03) |
|  | Rec voc 38M | 0.52(0.04) | <1x10^-10^ | 0.27(0.04) |
|  | Word form prod 38M | 0.57(0.04) | <1x10^-10^ | 0.32(0.04) |
|  | Combines words 38M | 0.16(0.04) | 4x10^-5^ | 0.03(0.01) |
| E4 | Morphology 38M | 0.86(0.02) | <1x10^-10^ | 0.74(0.04) |
|  | Rec voc 38M | -0.01(0.03) | 0.80 | 5x10^-5^(4x10^-4^) |
|  | Word form prod 38M | 0.09(0.03) | 0.01 | 0.01(0.01) |
|  | Combines words 38M | 0.20(0.04) | 6x10^-8^ | 0.04(0.02) |
| E5 | Rec voc 38M | 0.76(0.02) | <1x10^-10^ | 0.58(0.03) |
|  | Word form prod 38M | 0.05(0.03) | 0.10 | 2x10^-3^(3x10^-3^) |
|  | Combines words 38M | -0.08(0.03) | 0.03 | 0.01(0.01) |
| E6 | Word form prod 38M | 0.71(0.02) | <1x10^-10^ | 0.50(0.03) |
|  | Combines words 38M | 0.09(0.04) | 0.02 | 0.01(0.01) |
| E7 | Combines words 38M | 0.90(0.03) | <1x10^-10^ | 0.81(0.05) |

Cholesky decomposition of gross motor skills (6 months), self-care/symbolic actions (15 months), expressive vocabulary size (38 months), morphology (38 months), receptive vocabulary size (38 months), word form production (38 months) and the use of word combinations (38 months), in that order. The phenotypic covariance of the seven measures was dissected into five genomic (A1-A7) and seven non-genomic/residual factors (E1-E7). Analyses were based on all available observations for children across development (N≤6,978) and estimated with Genetic-relationship matrix structural equation modelling (grmsem, v.1.1.2) (Figure 5). Path coefficients (λ) and the corresponding variance explained (var) are shown with their respective standard errors in brackets. Individual-level data were retrieved from the Avon Longitudinal Study of Parents and Children.

Abbreviations: Exp, expressive; M, months; prod, production; rec, receptive; SE, standard error; var, variance; voc, vocabulary

## **Table S7: Developmental genetic relationships of gross motor behaviour (6 months) and self-care/symbolic actions (15 months) with language factor scores (sensitivity analysis)**

| **Factor** | **Trait** | **λ(SE)** | ***P*** | **var(SE)** |
| --- | --- | --- | --- | --- |
| A1 | Gross motor 6M | 0.41(0.07) | 8x10^-10^ | 0.17(0.06) |
|  | Self-care/symbolic 15M | 0.22(0.09) | 0.02 | 0.05(0.04) |
|  | Language FS_15M_ | 0.15(0.09) | 0.11 | 0.02(0.03) |
|  | Language FS_24M_ | 0.17(0.09) | 0.07 | 0.03(0.03) |
|  | Language FS_38M_ | 0.13(0.09) | 0.16 | 0.02(0.03) |
| A2 | Self-care/symbolic 15M | 0.38(0.07) | 4x10^-7^ | 0.14(0.06) |
|  | Language FS_15M_ | 0.26(0.09) | 4x10^-3^ | 0.08(0.06) |
|  | Language FS_24M_ | 0.28(0.10) | 4x10^-3^ | 0.07(0.05) |
|  | Language FS_38M_ | 0.30(0.10) | 2x10^-3^ | 0.08(0.05) |
| A3 | Language FS_15M_ | 0.19(0.10) | 0.07 | 0.05(0.06) |
|  | Language FS_24M_ | 0.16(0.15) | 0.28 | 0.02(0.05) |
|  | Language FS_38M_ | 0.10(0.17) | 0.54 | 0.05(0.06) |
| A4 | Language FS_24M_ | 0.28(0.07) | 3x10^-5^ | 0.02(0.04) |
|  | Language FS_38M_ | 0.14(0.10) | 0.16 | 0.005(0.03) |
| A5 | Language FS_38M_ | 0.16(0.11) | 0.14 | 0.03(0.06) |
| E1 | Gross motor 6M | 0.91(0.03) | <1x10^-10^ | 0.83(0.06) |
|  | Self-care/symbolic 15M | 0.17(0.04) | 1x10^-4^ | 0.03(0.01) |
|  | Language FS_15M_ | 0.21(0.04) | 8x10^-7^ | 0.01(0.01) |
|  | Language FS_24M_ | 0.15(0.04) | 8x10^-4^ | 0.004(0.01) |
|  | Language FS_38M_ | 0.10(0.04) | 0.02 | 0.03(0.01) |
| E2 | Self-care/symbolic 15M | 0.88(0.03) | <1x10^-10^ | 0.78(0.05) |
|  | Language FS_15M_ | 0.41(0.04) | <1x10^-10^ | 0.03(0.01) |
|  | Language FS_24M_ | 0.25(0.04) | 3x10^-9^ | 0.01(0.01) |
|  | Language FS_38M_ | 0.17(0.04) | 4x10^-5^ | 0.07(0.02) |
| E3 | Language FS_15M_ | 0.81(0.02) | <1x10^-10^ | 0.80(0.05) |
|  | Language FS_24M_ | 0.45(0.04) | <1x10^-10^ | 0.26(0.04) |
|  | Language FS_38M_ | 0.35(0.04) | <1x10^-10^ | 0.20(0.03) |
| E4 | Language FS_24M_ | 0.71(0.02) | <1x10^-10^ | 0.56(0.03) |
|  | Language FS_38M_ | 0.37(0.04) | <1x10^-10^ | 0.07(0.02) |
| E5 | Language FS_38M_ | 0.74(0.02) | <1x10^-10^ | 0.44(0.03) |

Cholesky decomposition of gross motor skills (6 months), self-care/symbolic actions (15 months), and the three estimated language factor scores (Language FS_15m_, Language FS_24m_ and Language FS_38m_), in that order. Language factor scores were estimated based on Pearson correlations and unstandardised parametrisation, as part of sensitivity analysis (Appendix S6). The phenotypic covariance of the five measures was dissected into five genomic (A1-A5) and five non-genomic/residual factors (E1-E5). Analyses were based on all available observations for children across development (N≤6,975) and estimated with Genetic-relationship matrix structural equation modelling (grmsem, v.1.1.2). Path coefficients (λ) and the corresponding variance explained (var) are shown with their respective standard errors in brackets. Individual-level data were retrieved from the Avon Longitudinal Study of Parents and Children.

Abbreviations: FS, factor score; M, months; SE, standard error; var, variance

## **Table S8: GREML and GRM-SEM SNP-heritability**

| **GRM-SEM model** | **Trait** | **GREML** | **GRM-SEM** |
| --- | --- | --- | --- |
|  |  | **SNP-h^2^(SE)** | **SNP-h^2^(SE)** |
| Language factor scores | Gross motor 6M | 0.18(0.06) | 0.17(0.06) |
|  | Self-care/symbolic 15M | 0.18(0.06) | 0.19(0.05) |
|  | Language FS_15M_ | 0.12(0.05) | 0.12(0.05) |
|  | Language FS_24M_ | 0.21(0.06) | 0.21(0.06) |
|  | Language FS_38M_ | 0.17(0.05) | 0.16(0.05) |
| Age-specific: 15 months | Gross motor 6M | 0.18(0.06) | 0.17(0.06) |
|  | Self-care/symbolic 15M | 0.18(0.06) | 0.19(0.05) |
|  | Exp voc 15M | 0.12(0.06) | 0.11(0.05) |
|  | Rec voc 15M | 0.08(0.05) | 0.07(0.05) |
| Age-specific: 24 months | Gross motor 6M | 0.18(0.06) | 0.17(0.05) |
|  | Self-care/symbolic 15M | 0.18(0.06) | 0.19(0.05) |
|  | Exp voc 24M | 0.16(0.06) | 0.17(0.06) |
|  | Morphology 24M | 0.09(0.06) | 0.21(0.06) |
|  | Combines words 24M | 0.16(0.06) | 0.11(0.06) |
| Age-specific:  38 months | Gross motor 6M | 0.18(0.06) | 0.17(0.05) |
|  | Self-care/symbolic 15M | 0.18(0.06) | 0.21(0.05) |
|  | Exp voc 38M | 0.13(0.06) | 0.15(0.05) |
|  | Morphology 38M | 0.05(0.06) | 0.15(0.05) |
|  | Rec voc 38M | 0.12(0.06) | 0.12(0.05) |
|  | Word form prod 38M | 0.11(0.05) | 0.15(0.05) |
|  | Combines words 38M | 0.16(0.06) | 0.11(0.05) |

SNP-heritability (SNP-h^2^) and corresponding standard errors (SE) were estimated with Genome-based Restricted Maximum-Likelihood (GREML) as implemented in Genome-wide Complex Trait Analysis (GCTA) software, and genetic-relationship-matrix structural equation modelling (GRM-SEM) for comparison, for all developmental genetic models.

Abbreviations: exp, expressive; FS, factor score; GCTA, Genome-wide Complex Trait Analysis; GREML, genome-based restricted maximum-likelihood; GRM-SEM, genetic-relationship-matrix structural equation modelling; M, months; prod, production; rec, receptive; SE, standard error; SNP-h^2^, var, variance; voc, vocabulary

# **Supplementary Figures**


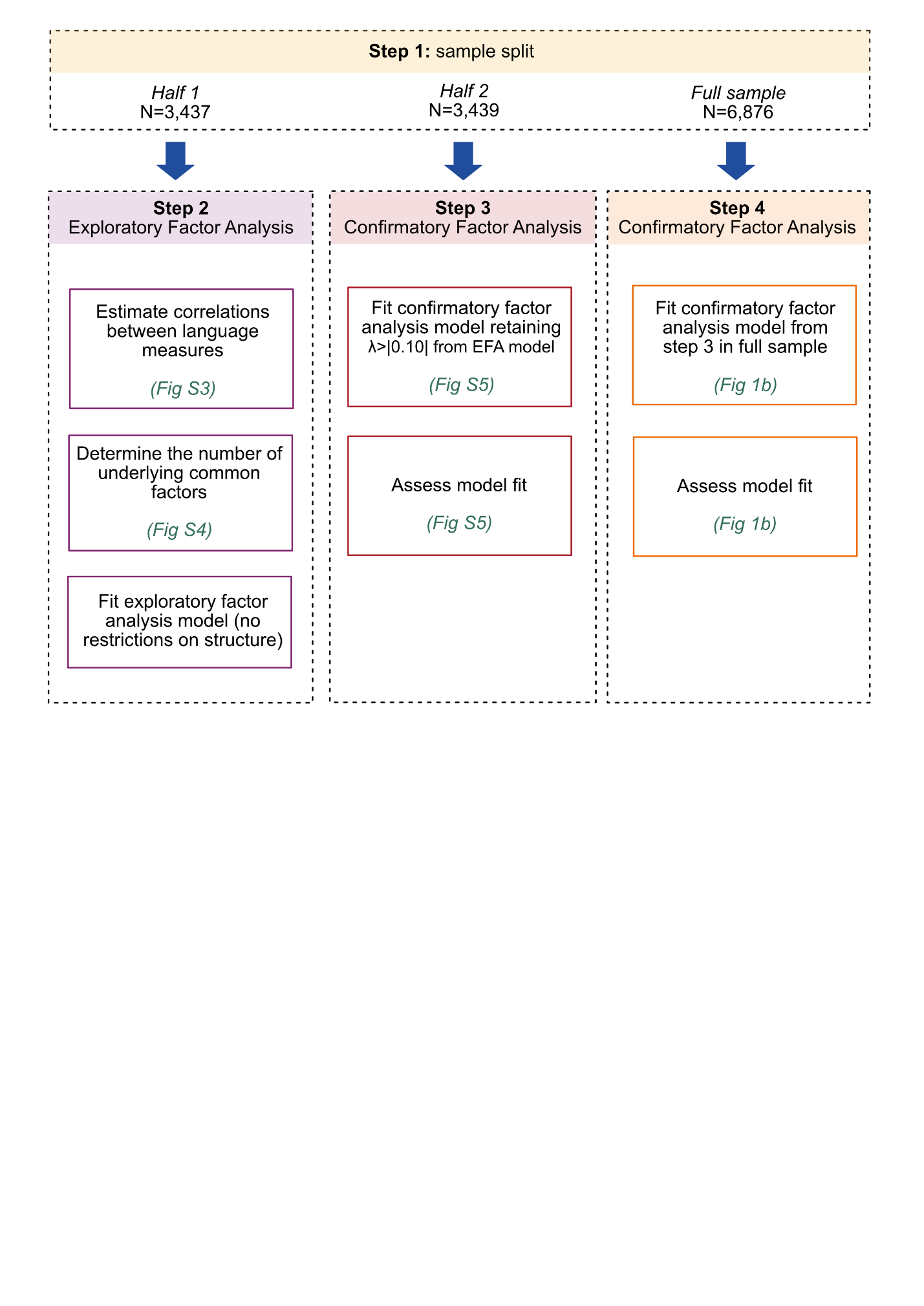


## **Figure S1: Exploratory and confirmatory factor analysis design**

As part of step one and following a split-half design, the ALSPAC data was randomly split into two independent halves (N_half1_=3,437; N_half2_=3,439) that were matched on age, sex and missingness (R:dplyr library, version 1.0.7). In the second step, the number of factors underlying the ten language measures was identified using eigenvalue decomposition of the Pearson correlation matrix based on the first half of the sample, and, subsequently, exploratory factor analysis was conducted. In step three, confirmatory factor analysis (CFA) was carried out in the second half of the sample and model fit was assessed. Finally, if CFA model fit was acceptable or good, the same structure was fitted to the full sample to increase statistical power in step 4.

Abbreviations: EFA, exploratory factor model; Fig, figure; N, sample size


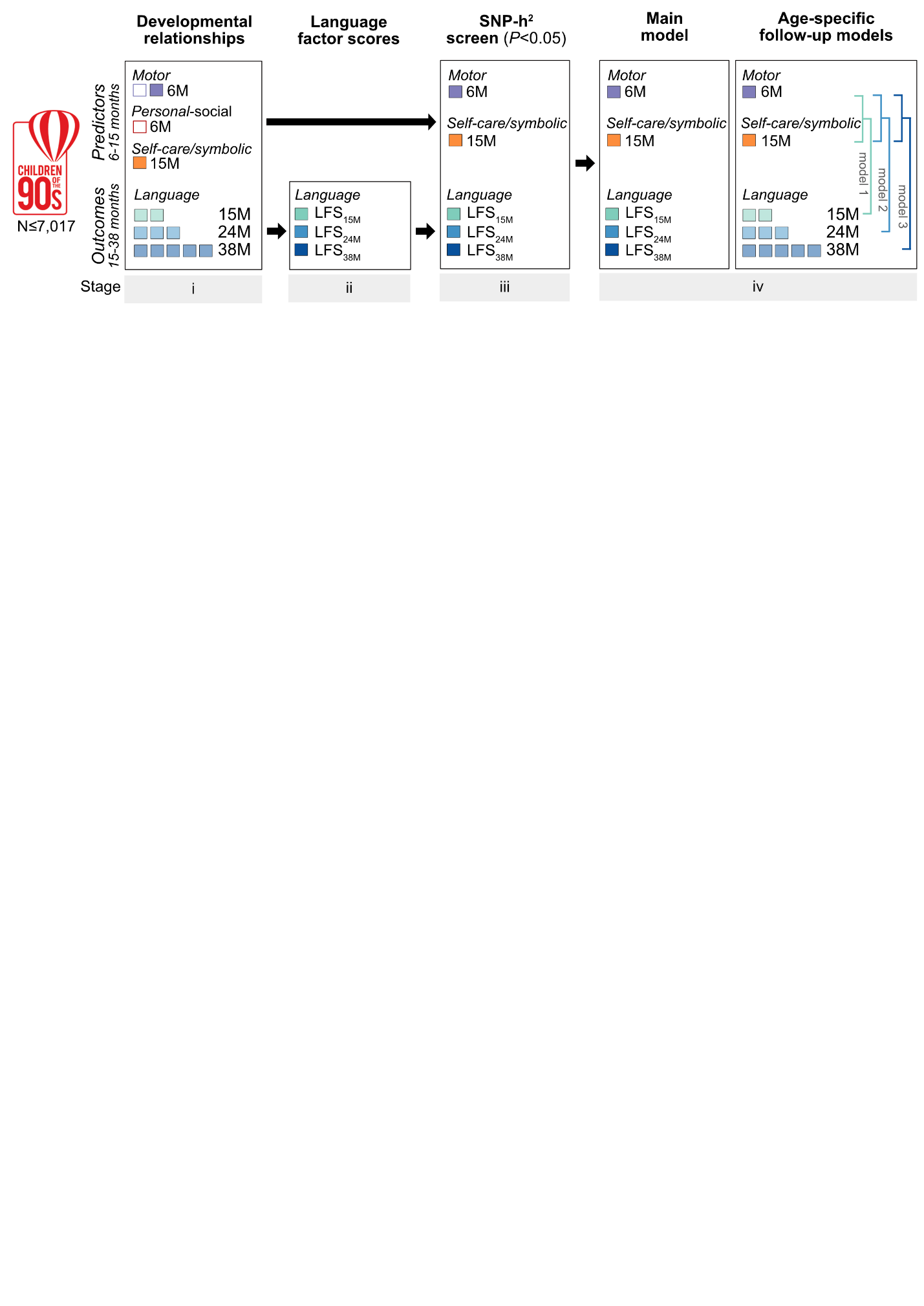


## **Figure S2: Study design**

Motor, personal-social, self-care/symbolic and language measures were studied in up to 7,017 children from the Avon Longitudinal Study of Parents and Children (ALSPAC) with genome-wide information available (Table 1). Adopting a multi-stage approach, we (i) studied evidence for developmental relationships, (ii) reduced the computational burden for downstream genetic analyses by combining language measures into overarching language factor scores (LFS), (iii) screened measures for Single-Nucleotide Polymorphism heritability (SNP-h^2^, *P*<0.05), and (iv) modelled underlying genomic and non-genomic relationships using genetic-relationship-matrix structural equation modelling (GRM-SEM)(St Pourcain et al., 2017). For step iv the main model consisted of both heritable predictors in combination with the three estimated language factor scores. Next, three age-specific follow-up models were fitted, each representing language measures that were highly related to one of the three identified overarching language factors.

Abbreviations: GRM-SEM, genetic-relationship-matrix structural equation modelling; LFS, language factor score; N, sample size; M, months; SNP-h^2^, Single-Nucleotide Polymorphism heritability


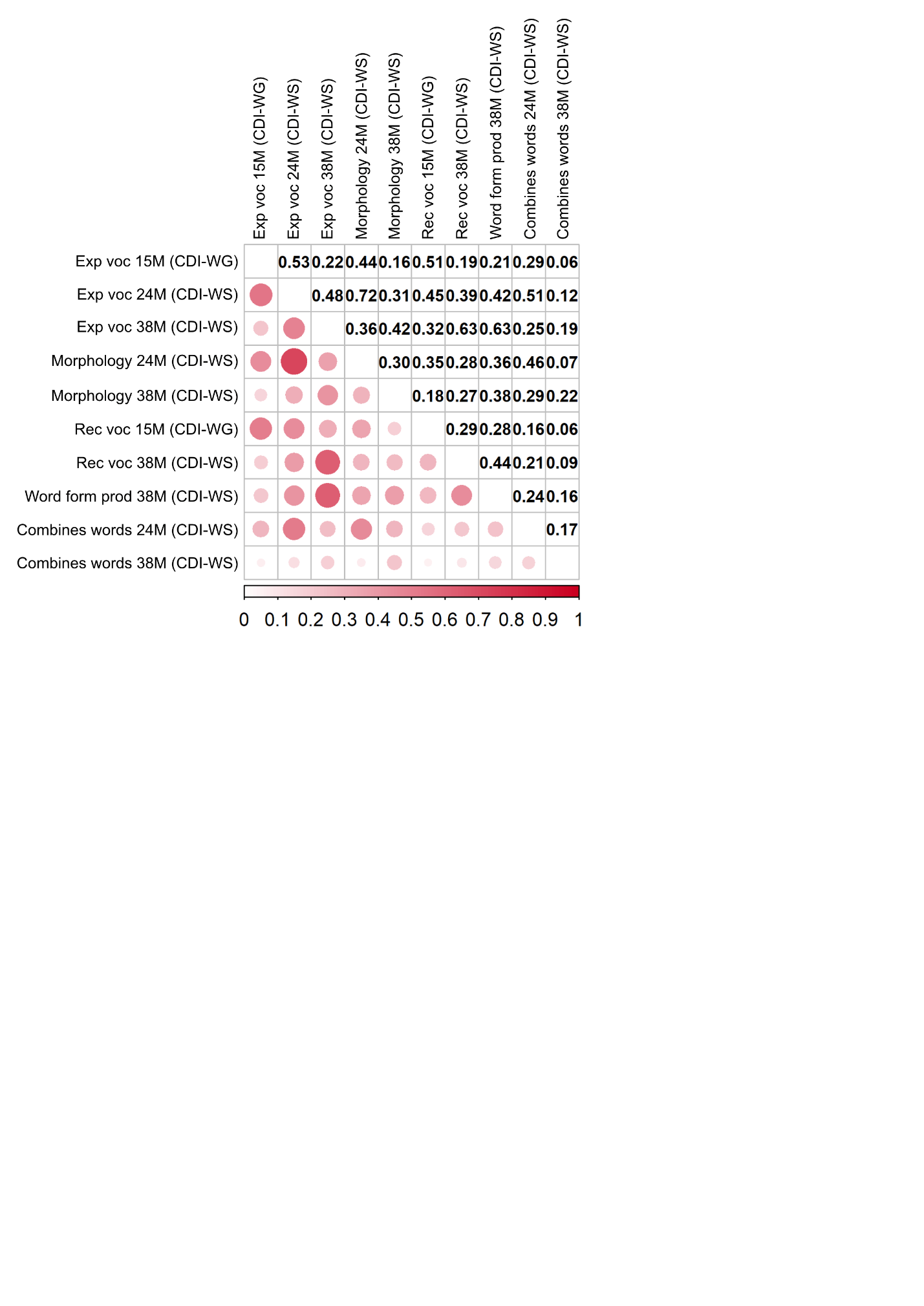


## **Figure S3: EFA: Phenotypic correlations among language measures**

Phenotypic correlations among fully-adjusted two-stage transformed language measures (continuous scores) and centred and scaled deviance residuals (binary scores) were estimated using Pearson’s correlations in the first half of the sample (N=3,437).

Abbreviations: CDI-WG, Communicative Development Inventories – Words and Gestures; CDI-WS; Communicative Development Inventories – Words and Sentences; Exp, expressive; M, months; Morph, morphology; prod, production; Rec, receptive; voc, vocabulary


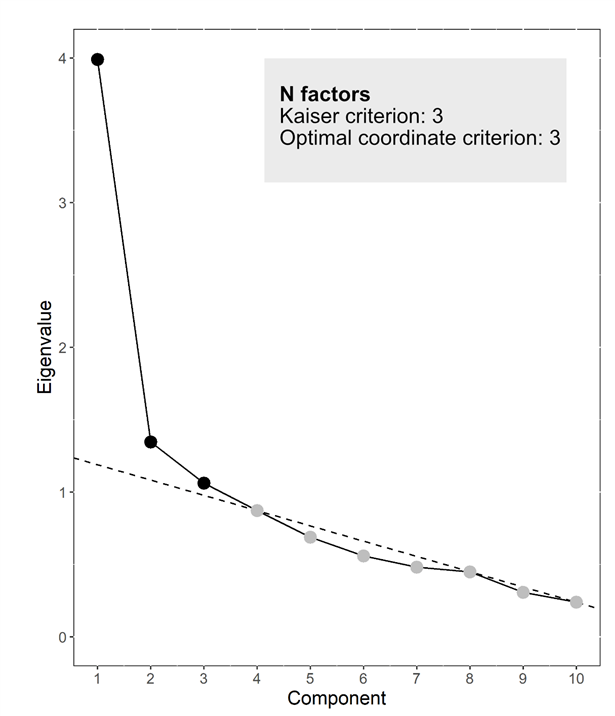


## **Figure S4: Estimated number of common factors underlying language measures**

Scree plots based on the eigenvalue decomposition of phenotypic correlations among language measures derived using Pearson correlations. The number of estimated common underlying factors (in black) according to the Kaiser and optimal coordinate criterion. The dashed line indicates the “scree” of the plot (grey points).


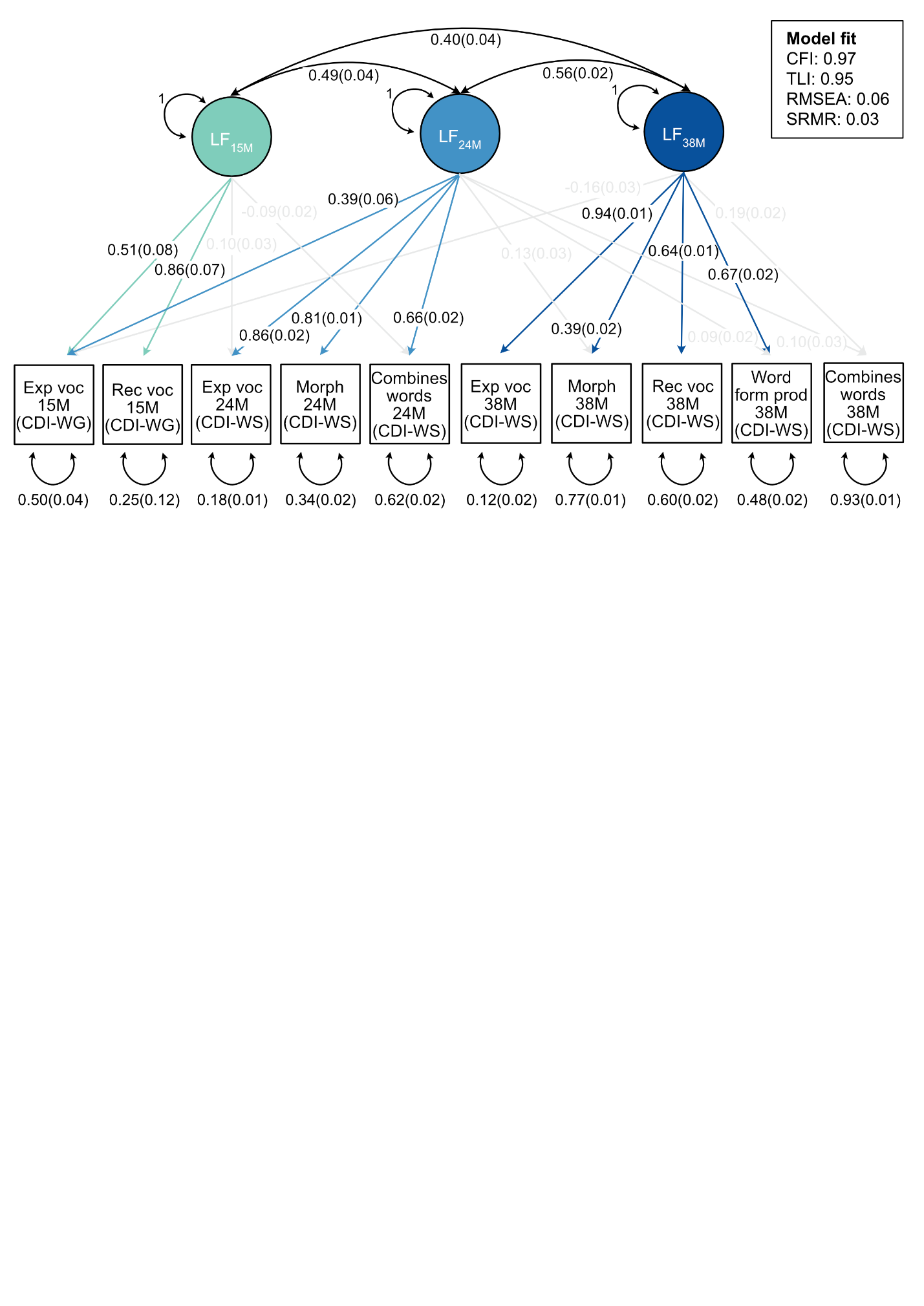


## **Figure S5: Confirmatory factor analysis model (split-half design)**

Confirmatory factor analysis model across the ten language abilities as estimated with lavaan following standardised parametrisation based on the second half of the sample (N=3,439). Fully standardised factor loadings (λ) are shown for *P*<0.05 only, with meaningful factor loadings (i.e. λ ≥ |0.30|) depicted in colour. Observed measures are represented by squares and factors by circles. Single-headed arrows (paths) define relationships between variables. Language factors (LF) and their factor loadings are labelled according to the language abilities with the strongest factor loadings. Double headed arrows between factors depict correlations. Double-headed arrows at each LF reflect its variance, which has been constrained to unit variance (standardised parametrisation). Double-headed arrows at each observed measure reflect its residual variance.

Abbreviations: CDI-WG, Communicative Development Inventories – Words and Gestures; CDI-WS; Communicative Development Inventories – Words and Sentences; CFI, comparative fit index; Exp, expressive; LF, language factor; M, months; Morph, morphology; prod, production; Rec, receptive; RMSEA, root mean square error of approximation; SRMR; standardized root mean squared residual; TLI, Tucker-Lewis index; voc, vocabulary


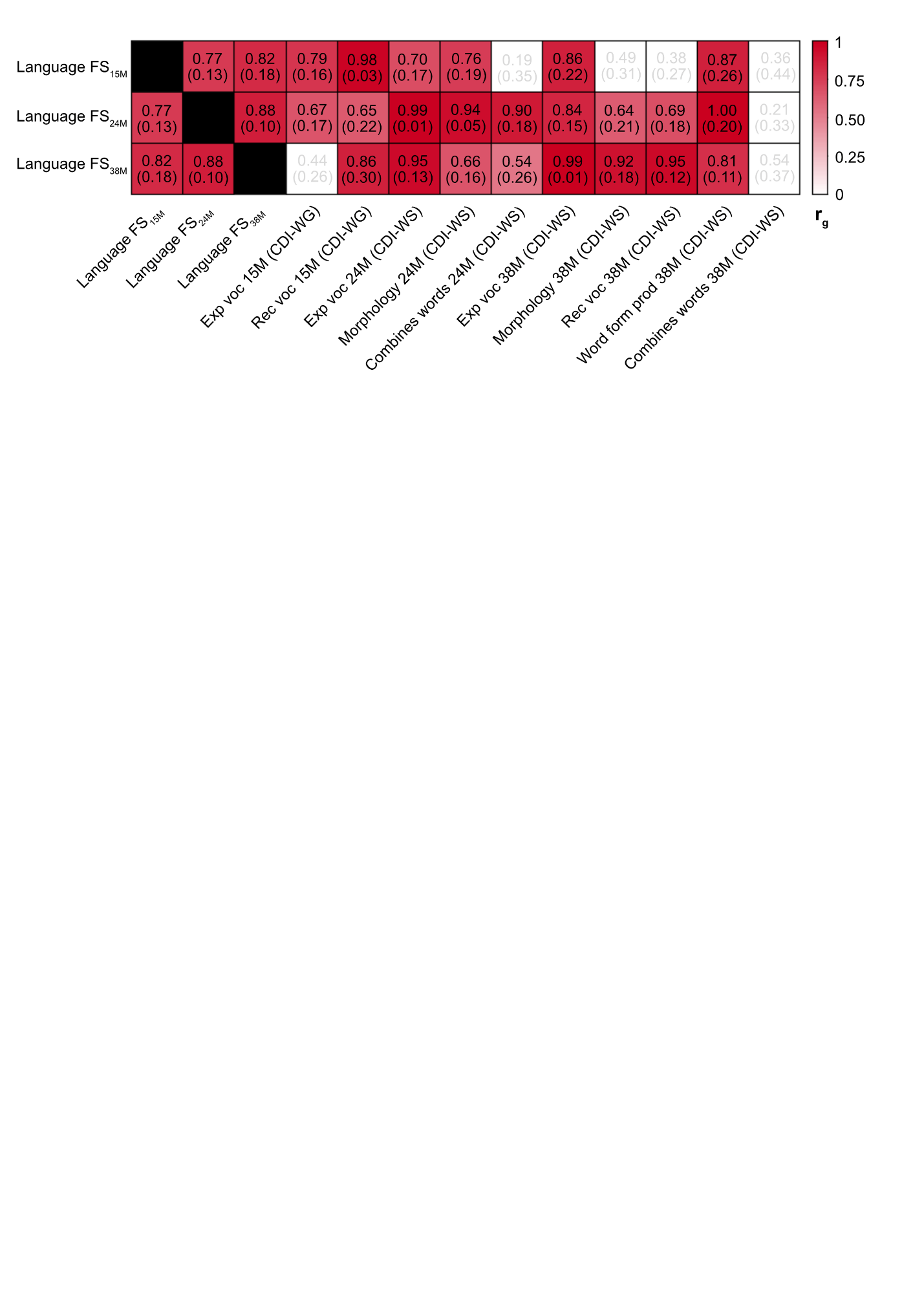


## **Figure S6: Genetic correlations of language factor scores with individual language measures**

Genetic correlations (r_g_) were estimated with bivariate Genome-based Restricted Maximum-Likelihood (GREML) as implemented in Genome-wide Complex Trait Analysis (GCTA) software, based on directly genotyped SNPs, for all ten language measures and the three estimated language factor scores (Language FS). Bars represent standard errors. Genetic correlations with *P*<0.05 are shown in black on a red background, while genetic correlations with *P*≥0.05 are shown in grey on a white background.

Abbreviations: CDI-WG, Communicative Development Inventories – Words and Gestures; CDI-WS; Communicative Development Inventories – Words and Sentences; DDST, Denver Developmental Screening Test; exp, expressive; FS, factor score; M, months; Morph, morphology; prod, production; rec, receptive; voc, vocabulary


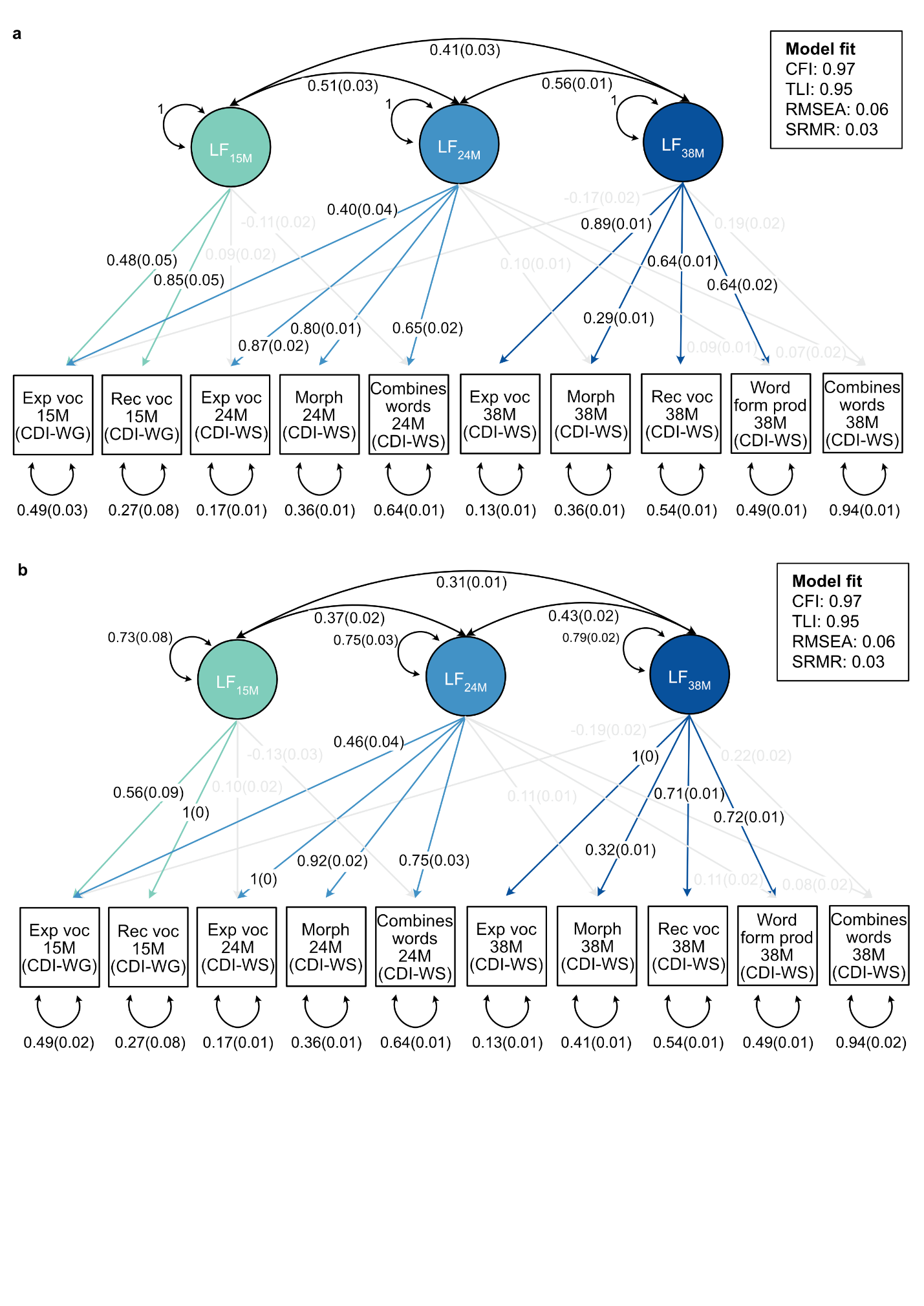


## **Figure S7: Factor structure underlying language abilities (sensitivity analysis)**

Confirmatory factor analysis model across the ten language abilities as estimated with lavaan following (a) standardised parametrisation and (b) unstandardised parametrisation, both based on the full sample (N=6,876). Unstandardised factor loadings (λ) are shown for *P*<0.05 only, with λ ≥ |0.30| depicted in colour. Observed measures are represented by squares and factors by circles. Single-headed arrows (paths) define relationships between variables. Language factors (LF) and their factor loadings are labelled according to the language abilities with the strongest factor loadings. Double headed arrows between factors depict correlations. Double-headed arrows at each LF reflect its variance. Double-headed arrows at each observed measure reflect its residual variance.

Abbreviations: CDI-WG, Communicative Development Inventories – Words and Gestures; CDI-WS; Communicative Development Inventories – Words and Sentences; CFI, comparative fit index; Exp, expressive; LF, language factor; M, months; Morph, morphology; prod, production; Rec, receptive; RMSEA, root mean square error of approximation; SRMR; standardized root mean squared residual; TLI, Tucker-Lewis index; voc, vocabulary


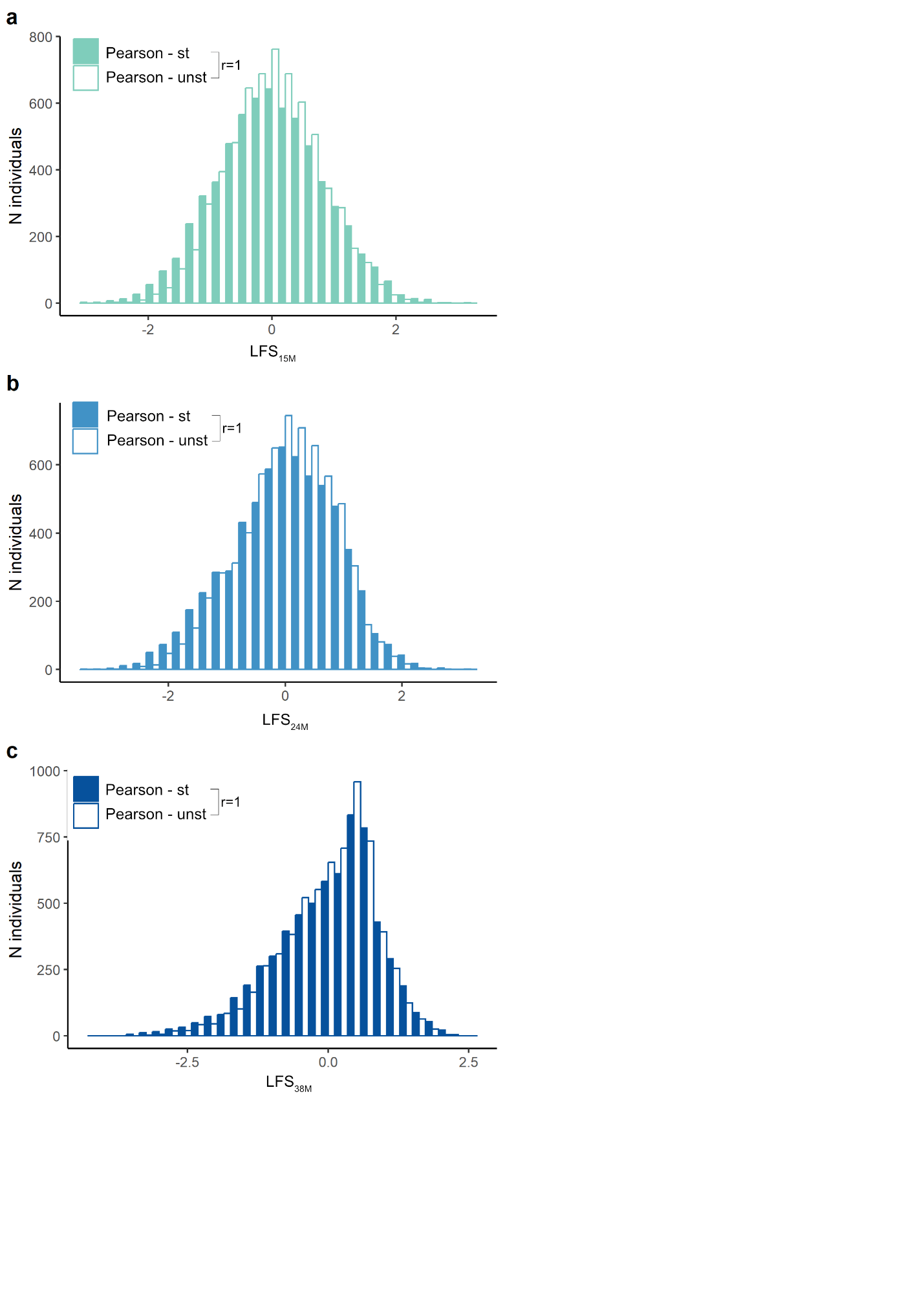


## **Figure S8: Distribution of derived language factor scores**

Language factor scores (LFS) were derived with lavaan using the regression predictor for (a) LFS_15M_, (b) LFS_24M_ and (c) LFS_38M_, all following standardised and unstandardised parametrisation. Correlations between derived language factor scores were computed using pair-wise complete observations and Pearson correlation.

Abbreviations: LFS, language factor score; N, sample size; r, Pearson correlation coefficient; stand, standardised parametrisation; unstand, unstandardised parametrisation

# **Supplementary References**

Bentler, P. M. (1990). Comparative Fit Indexes in Structural Models, *107*(2).

Bentler, P. M. (1995). EQS 5.

Bleses, D., Vach, W., Slott, M., Wehberg, S., Thomsen, P., Madsen, T. O., & Basbøll, H. (2008). The Danish Communicative Developmental Inventories: Validity and main developmental trends. *Journal of Child Language*, *35*(3), 651–669.

Boyd, A., Golding, J., Macleod, J., Lawlor, D. A., Fraser, A., Henderson, J., Molloy, L., et al. (2013). Cohort Profile: The ’children of the 90s’--the index offspring of the Avon Longitudinal Study of Parents and Children. *International Journal of Epidemiology*, *42*(1), 111–127.

Clapp Sullivan, M. L., Schwaba, T., Harden, K. P., Grotzinger, A. D., Nivard, M. G., & Tucker-Drob, E. M. (2024). Beyond the factor indeterminacy problem using genome-wide association data. *Nature Human Behaviour*, *8*(2), 205–218.

Dale, P. S. (1991). The validity of a parent report measure of vocabulary and syntax at 24 months. *Journal of Speech and Hearing Research*, *34*(3), 565–571.

Devlieger, I., Mayer, A., & Rosseel, Y. (2016). Hypothesis Testing Using Factor Score Regression: A Comparison of Four Methods. *Educational and Psychological Measurement*, *76*(5), 741–770. SAGE Publications Inc.

Dionne, G., Dale, P. S., Boivin, M., & Plomin, R. (2003). Genetic Evidence for Bidirectional Effects of Early Lexical and Grammatical Development. *Child Development*, *74*(2), 394–412.

van Eldik, M. C. M., Schlichting, J. E. P. T., Iutje Spelberg, H. C., van der Meulen, B. F., & van der Meulen, Sj. (2004). *Reynell Test voor Taalbegrip: Handleiding* (4e herziene druk.). Amsterdam: Harcourt Test Publishers.

Fenson, L., Dale, P., Reznick, J. S., Thal, D., Bates, E., Hartung, J., Pethick, S., et al. (1993). *User’s Guide and Technical Manual for the MacArthur Communicative Development Inventories*. San Diego: Singular Publishing.

Fenson, L., Pethick, S., Renda, C., Cox, J. L., Dale, P. S., & Reznick, J. S. (2000). Short-form versions of the MacArthur Communicative Development Inventories. *Applied Psycholinguistics*, *21*(1), 95–116.

Frank, M. C., Braginsky, M., Yurovsky, D., & Marchman, V. A. (2021). *Variability and Consistency in Early Language Learning: The Wordbank Project*. Cambridge: MIT Press.

Frankenburg, W. K., & Dodds, J. B. (1967). The Denver Developmental Screening Test. *The Journal of Pediatrics*, *71*(2), 181–191.

Frankenburg, W. K., Goldstein, A. D., & Camp, B. W. (1971). The revised Denver Developmental Screening Test: Its accuracy as a screening instrument. *The Journal of Pediatrics*, *79*(6), 988–995.

Fraser, A., Macdonald-Wallis, C., Tilling, K., Boyd, A., Golding, J., Davey Smith, G., Henderson, J., et al. (2013). Cohort Profile: The Avon Longitudinal Study of Parents and Children: ALSPAC mothers cohort. *International Journal of Epidemiology*, *42*(1), 97–110.

Gorsuch, R. L. (1983). *Factor Analysis, 2nd Edition* (2nd edition.). Hillsdale, N.J: Lawrence Erlbaum Associates.

Houston-Price, C., Mather, E., & Sakkalou, E. (2007). Discrepancy between parental reports of infants’ receptive vocabulary and infants’ behaviour in a preferential looking task. *Journal of Child Language*, *34*(4), 701–724.

Hu, L., & Bentler, P. M. (1999). Cutoff criteria for fit indexes in covariance structure analysis: Conventional criteria versus new alternatives. *Structural Equation Modeling: A Multidisciplinary Journal*, *6*(1), 1–55. Routledge.

Hu, L.-T., & Bentler, P. M. (1995). Evaluating model fit. *Structural equation modeling: Concepts, issues, and applications* (pp. 76–99). Thousand Oaks, CA, US: Sage Publications, Inc.

Kaiser, F. (1960). The Application of Electronic Computers to Factor Analysis, *20*(1).

Kaplan, D. W. (2008). *Structural Equation Modeling: Foundations and Extensions* (2nd edition.). Los Angeles: SAGE Publications, Inc.

Law, J., & Roy, P. (2008). Parental Report of Infant Language Skills: A Review of the Development and Application of the Communicative Development Inventories. *Child and Adolescent Mental Health*, *13*(4), 198–206.

Lee, S. H., Yang, J., Goddard, M. E., Visscher, P. M., & Wray, N. R. (2012). Estimation of pleiotropy between complex diseases using single-nucleotide polymorphism-derived genomic relationships and restricted maximum likelihood. *Bioinformatics (Oxford, England)*, *28*(19), 2540–2542.

Marchman, V. A., Dale, P. S., & Fenson, L. (2023). *MacArthur-Bates Communicative Development Inventories: User’s Guide and Technical Manual* (Third.). Paul H. Brookes Publishing Company.

van Noort-van der Spek, I. L., Franken, M.-C. J. P., Swarte, R. M. C., & Weisglas-Kuperus, N. (2021). Validity of an early parent-report questionnaire for language disorder in very preterm children from 2 to 10 years of age. *European journal of paediatric neurology: EJPN: official journal of the European Paediatric Neurology Society*, *34*, 1–6.

Price, A. L., Patterson, N. J., Plenge, R. M., Weinblatt, M. E., Shadick, N. A., & Reich, D. (2006). Principal components analysis corrects for stratification in genome-wide association studies. *Nature Genetics*, *38*(8), 904–909.

Purcell, S., Neale, B., Todd-Brown, K., Thomas, L., Ferreira, M. A. R., Bender, D., Maller, J., et al. (2007). PLINK: A Tool Set for Whole-Genome Association and Population-Based Linkage Analyses. *American Journal of Human Genetics*, *81*(3), 559–575.

Ring, E. D., & Fenson, L. (2000). The correspondence between parent report and child performance for receptive and expressive vocabulary beyond infancy. *First Language*, *20*(59), 141–159.

Rosseel, Y. (2012). lavaan: An R Package for Structural Equation Modeling. *Journal of Statistical Software*, *48*, 1–36.

Ruscio, J., & Roche, B. (2012). Determining the number of factors to retain in an exploratory factor analysis using comparison data of known factorial structure. *Psychological Assessment*, *24*(2), 282–292.

Shapland, C. Y., Verhoef, E., Davey Smith, G., Fisher, S. E., Verhulst, B., Dale, P. S., & St Pourcain, B. (2021). Multivariate genome-wide covariance analyses of literacy, language and working memory skills reveal distinct etiologies. *Npj Science of Learning*, *6*(1), 1–12.

Sodini, S. M., Kemper, K. E., Wray, N. R., & Trzaskowski, M. (2018). Comparison of Genotypic and Phenotypic Correlations: Cheverud’s Conjecture in Humans. *Genetics*, *209*(3), 941–948.

Sofer, T., Zheng, X., Gogarten, S. M., Laurie, C. A., Grinde, K., Shaffer, J. R., Shungin, D., et al. (2019). A fully adjusted two-stage procedure for rank-normalization in genetic association studies. *Genetic Epidemiology*, *43*(3), 263–275.

St Pourcain, B., Eaves, L. J., Ring, S. M., Fisher, S. E., Medland, S., Evans, D. M., & Smith, G. D. (2017). Developmental changes within the genetic architecture of social communication behaviour: A multivariate study of genetic variance in unrelated individuals. *Biological Psychiatry*, *83*, 598–606.

Steiger, J. H. (1990). Structural Model Evaluation and Modification: An Interval Estimation Approach. *Multivariate Behavioral Research*, *25*(2), 173–180.

Tucker, L. R., & Lewis, C. (1973). A reliability coefficient for maximum likelihood factor analysis. *Psychometrika*, *38*(1), 1–10.

Verhoef, E., Shapland, C. Y., Fisher, S. E., Dale, P. S., & St Pourcain, B. (2020). The developmental origins of genetic factors influencing language and literacy: Associations with early-childhood vocabulary. *Journal of Child Psychology and Psychiatry*.

Verhoef, E., Shapland, C. Y., Fisher, S. E., Dale, P. S., & St Pourcain, B. (2021). The developmental genetic architecture of vocabulary skills during the first three years of life: Capturing emerging associations with later-life reading and cognition. *PLOS Genetics*, *17*(2), e1009144.

Verhulst, B., & Estabrook, R. (2012). Using genetic information to test causal relationships in cross-sectional data. *Journal of Theoretical Politics*, *24*(3), 328–344.

Yang, J., Lee, S. H., Goddard, M. E., & Visscher, P. M. (2011). GCTA: A tool for genome-wide complex trait analysis. *American Journal of Human Genetics*, *88*(1), 76–82.
